# Absolute quantification of fluorescent protein fusions by mass spectrometry

**DOI:** 10.1002/pro.70556

**Published:** 2026-04-06

**Authors:** Anna Shevchenko, Archishman Ghosh, Andrea Schuhmann, Aliona Bogdanova, Henrik Thomas, Viditha Rao, Eric R. Geertsma, T.‐Y. Dora Tang, Andrej Shevchenko

**Affiliations:** ^1^ MPI of Molecular Cell Biology and Genetics Dresden Germany; ^2^ Department of Synthetic Biology Universität des Saarlandes Campus, Gebaude B2.2 Saarbrücken 66123 Germany

**Keywords:** absolute quantification of proteins, cell‐free expression systems, fluorescent chromophores maturation, fusions with fluorescent proteins, kinetics of protein expression, quantitative mass spectrometry

## Abstract

Fusions with fluorescent proteins (FPs) play a pivotal role in experimental biology because of their sensitive and spatially precise visualization by spectroscopy. However, observed fluorescence is not always proportional to their molar concentration. Only a fraction of the fusion protein that contains the mature fluorescence chromophore is detectable by spectroscopy and there is no accurate and generic method for estimating its molar abundance. We have developed a fluorescence‐independent mass spectrometry‐based method for absolute (molar) sub‐femtomole quantification of FP‐fusions that also estimates the fraction of fully matured chromophore. The method exploits an isotopically labeled 68 kDa recombinant protein standard expressed in *E. coli* and used without further purification. This chimeric protein contains multiple peptide proxies for six prototypical FPs (mCherry, mScarlet‐I, mKate2, EGFP, mNeonGreen, and Dendra2) and two self‐labelling (Halo‐ and SNAP‐) tags and supports the quantification of proteins fused to any of 615 common FPs and tags. The method can be used broadly for the absolute quantification of fluorescent fusions *in vivo* and *in vitro* and is complementary to fluorescence measurements. We further combined mass spectrometry with fluorescence spectroscopy to study expression kinetics of FP fusions in cell‐free systems. Molar concentrations of the expressed fusion, its fraction with mature chromophore, and of the fluorescing protein were integrated into a mathematical model to obtain kinetic rates of translation, chromophore maturation, and folding.

## 1 INTRODUCTION

Fluorescent proteins (FPs) revolutionized molecular and cell biology by providing a visual tool to track proteins *in vitro* and *in vivo*. Their discovery and development were recognized by the Nobel Prize awarded to O. Shimomura, M. Chalfie, and R. Y. Tsien in 2008 (Swaminathan 2009). The FP family comprises several hundred natural and designed proteins (see http://www.fpbase.com, FPBase 2026) each having distinctive photochemical (e.g., excitation and emission maxima or brightness) and physicochemical (e.g., lifetime, photostability, or maturation time) characteristics (Cranfill et al. 2016; Drepper et al. 2007; Miyawaki et al. 2005; Zhang et al. 2023) and the potential for engineering novel, application‐specific FPs remains significant (Rodriguez et al. 2017). To enhance optical detectability and imaging properties, a family of self‐labelling protein tags (e.g., Halo, SNAP, or CLIP) has been developed alongside prototypical FPs. While these tags themselves are not fluorescent, they covalently bind small fluorescent dyes (Gautier et al. 2008; Keppler et al. 2003; Los and Wood 2007), which exhibit superior brightness and photostability.

Fusions of FP to non‐fluorescent proteins (further termed as “FP‐fusions”) or to specific recognition domains are utilized in a variety of applications. FP‐fusions serve as markers of protein localization, indicators of gene expression and biosensors for non‐invasive, spatial and temporal imaging of biochemical processes and metabolites in living systems (Chudakov et al. 2010; Ibraheem and Campbell 2010; Collection 2021). They can be visualized in cells, organelles, or even smaller sub‐cellular structures by means of ultrahigh‐resolution fluorescence microscopy with extraordinary specificity and spatial precision (reviewed in Combs and Shroff 2017 and Prakash et al. 2022). However, the observed fluorescent signal provided by the genetically encoded FP‐reporter is not always proportional to the molar abundance of FP‐fusion (Geertsma et al. 2008). In particular, in order to become fluorescent, the chromophore in an FP sequence (with the exception of non‐prototypical group of FPs) (Drepper et al. 2007; Kumagai et al. 2013; Roldan‐Salgado et al. 2022; Shu et al. 2009) matures via cyclization, oxidation, and dehydration of three successively positioned amino acid residues. This chromophore‐forming triad comprising the amino acids sequence of …(M/Q/G/H)YG… in green and red FPs or another aromatic amino acid residue at the second position in blue FPs should yield a conjugated cyclic structure (Craggs 2009; Goedhart et al. 2012; Subach and Verkhusha 2012). Completing the chromophore maturation may take anywhere from minutes to hours (Balleza et al. 2018). The observed fluorescence could be affected by the intrinsic properties of FP‐fusion and by experiment‐dependent factors including photobleaching, blinking, ligands binding, or multimerization, to mention only a few. Hence, a significant (conveniently termed as “dark”) fraction of full‐length FP‐fusions may not be able to absorb or emit light because it lacks the correctly assembled chromophore. For self‐labelling tags quantitative readout could be compromised by limited labelling efficiency as well as hindered delivery, unspecific binding or incomplete folding of ligands (Jung et al. 2013).

There is considerable interest in achieving robust and reproducible molar quantification of FP‐fusions (Cho et al. 2022; Csibra and Stan 2022; Levy et al. 2014; Prakash et al. 2022; Rodriguez et al. 2017). Knowledge of the molar amount of FP‐fusion enables direct comparison with endogenous protein levels and facilitates the analysis of a protein's interaction stoichiometry, kinetics of protein expression, folding and turnover in vivo and in vitro. Complementing fluorescent spectroscopy with absolute quantification of FP‐fusions by mass spectrometry will ensure the analytical rigor necessary for a broad spectrum of biological applications.

Absolute (molar) quantification of proteins by mass spectrometry typically relies on the known amount of isotopically labeled peptide standards spiked into proteins digest prior LC–MS/MS analysis (reviewed in Kito and Ito 2008, Ankney et al. 2018, Calderon‐Celis et al. 2018, Rozanova et al. 2021, and Rusilowicz et al. 2023). The peptide standards can be chemically synthesized or produced by in‐situ enzymatic cleavage of metabolically labeled protein chimeras comprising concatenated peptide proxies for multiple target proteins (Bennett et al. 2017; Beynon et al. 2005; Gerber et al. 2003; Kito et al. 2016; Kumar et al. 2018; Rzagalinski et al. 2022). While effective, this approach is cumbersome and inflexible because for each target protein several unique peptides should be selected, synthesized, and evaluated individually. Furthermore, when applied to FP‐fusions, such analyses can only determine their total amount, while the proportion of the “dark” fraction, lacking a mature chromophore, remains unknown.

Here we demonstrate that absolute quantification of FP‐fusions can be performed by mass spectrometry using a single generic chimeric protein standard containing peptide proxies of prototypical FPs and self‐labelling tags. The same analysis could determine the molar fraction of FPs having the mature chromophore structure. We show that in cell‐free systems the combination of quantitative mass spectrometry and fluorescence spectroscopy reveals the kinetics of expression of FP fusions and maturation of their fluorescent chromophore, which could be described by a generic sequential reaction model.

## 2 RESULTS AND DISCUSSION

### 2.1 Design of chimeric protein standard and quantification workflow

Within a protein fusion, FP or self‐labelling protein tag is genetically encoded to the protein of interest in a known (typically, equimolar) stoichiometric ratio. We reasoned that the peptides originating from the FP part of the fusion could proxy the molar quantification of the entire protein construct. Conceivably, it should be possible to quantify any FP‐fusion using a single set of proxy peptides representing frequently used FPs.

**FIGURE 1:**
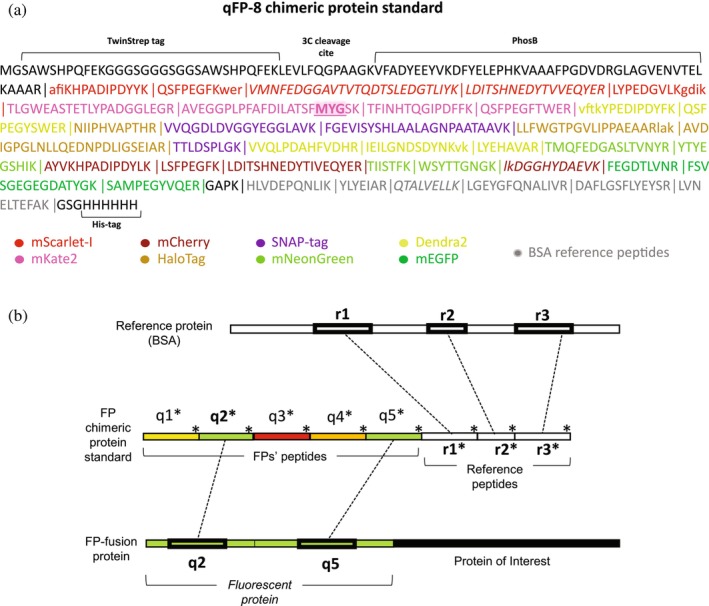
Absolute quantification of FP‐fusions using qFP‐8 standard. (a) Sequence and modular composition of qFP‐8. Peptide proxies from FPs and BSA reference peptides are color coded; tryptic peptides are spaced by vertical lines. Sequences extending some quantotypic peptides are in lower case. Peptides in italic did not pass validation check and were not used for quantification. The chromophore tripeptide […MYG…] within one of the mKate2 peptides is shadowed. (b) The scheme explaining the quantification workflow: q1…q5 represent (in this example, five) FP peptide proxies, colors indicate proxies of different FPs; r1…r3 are reference peptides identical to peptides from BSA. Asterisks indicate qFP‐8 peptides metabolically labeled with ^13^C_6_^15^N_4_‐Arg or ^13^C_6_‐Lys. To exemplify the workflow, green FP is shown as fused to the N‐terminus of a protein of interest.

To select the peptide proxies we first analyzed tryptic digests of six prototypical FPs: the constitutively fluorescent red mCherry and mScarlet‐I; the far‐red mKate2; the green mEGFP; the yellow‐green mNeonGreen; and the photoconvertible green‐to‐red Dendra2 (see Table S1, and FPbase resource for details), and the self‐labelling Halo‐ and SNAP‐Tag proteins (Keppler et al. 2003; Los and Wood 2007)—for brevity, here we will collectively term them as FPs. In LC–MS/MS chromatograms we picked abundant, consistently detectable fully tryptic peptides whose sequences (preferably, but not necessarily) contained no Cys or Met, and no N‐terminal Glu or Asp residues (Slechtova et al. 2015) that served as quantotypic peptide candidates (reviewed in Simpson and Beynon 2012). To leverage incomplete tryptic cleavage, we further extended the sequences of some candidate peptides by 2–3 amino acid residues to mimic corresponding sequence stretches in the source FPs (Whiteaker et al. 2021). Taken together, we selected 31 quantotypic peptide candidates consisting of 7–26 amino acid residues such that each of the eight FPs was represented by 3–5 unique sequences. Next, we produced a metabolically labeled chimeric protein termed qFP‐8 (for **q**uantification of **8** **FP**s) that comprised these 31 peptides together with 6 peptides from the reference protein (BSA) and 4 peptides from an alternative reference protein (PhosB) flanked by sequences of Strep‐ and His‐tags; see Kumar et al. (2018) for details on its design and expression in the *Δarg1Δlys1* strain of *E. coli*, metabolic labelling with ^13^C_6_^15^N_4_‐Arg and ^13^C_6_‐Lys, and quality control by LC–MS/MS (Figures 1a and S1). It was designed as a generic internal standard that enables absolute quantification of any protein fused to any individual FP sharing quantotypic peptides with the qFP‐8 sequence (Table S2). No protein‐specific standards are required and proteins fused to different FPs could be quantified in parallel.

To quantify FP‐fusions, we adapted the workflow based on the MS Western and FastCAT protocols (Kumar et al. 2018; Rzagalinski et al. 2022) (Figures 1b and S2). Briefly, FP‐fusions, metabolically labeled qFP‐8 and a known amount of reference protein (BSA) were co‐digested with trypsin and the digest was analyzed by LC–MS/MS. Since chimeric protein standards (including qFP‐8) are highly expressed in *E. coli*, there is no need for their purification. Appropriate aliquots of whole cell lysate can be directly used in the analyses (Kumar et al. 2018; Rzagalinski et al. 2022; Skiba et al. 2023). By comparing areas of XIC peaks of unlabeled reference peptides (from BSA) and corresponding labeled peptides (from qFP‐8), we determined the exact amount of co‐digested qFP‐8 and, consequently, of all quantotypic peptides encoded within the qFP‐8 sequence. Pairs of labeled (from qFP‐8) and unlabeled (from FP fusion) peptide proxies served as independent single‐point calibrants for quantifying the molar abundance of the FP moiety and, in turn, the entire FP‐fusion.

### 2.2 Validation of absolute quantification of FP‐fusions

Quantification of FP‐fusion follows the extensively validated MS Western protocol (Kumar et al. 2018). However, both MS Western and its more advanced variant FastCAT (Rzagalinski et al. 2022) rely on quantotypic peptides picked by LC–MS/MS analyses of tryptic digests of full‐length target proteins. We wondered if FP peptide proxies can accurately reflect the abundance of full‐length FP‐fusions that could contain peptides with superior ionization properties. Since no FP‐fusion standards with exactly known concentration and guaranteed purity are commercially available, we validated the quantification protocol in two ways. First, we tested if the relative abundance of FP peptide proxies in the digests of FP‐fusions and of qFP‐8 standard was the same. Second, we checked if the targeted quantification using qFP‐8 peptides corroborates independent LC–MS/MS determinations based on other (and, potentially, more abundant) peptides originating from the entire sequence of FP‐fusions.

**FIGURE 2:**
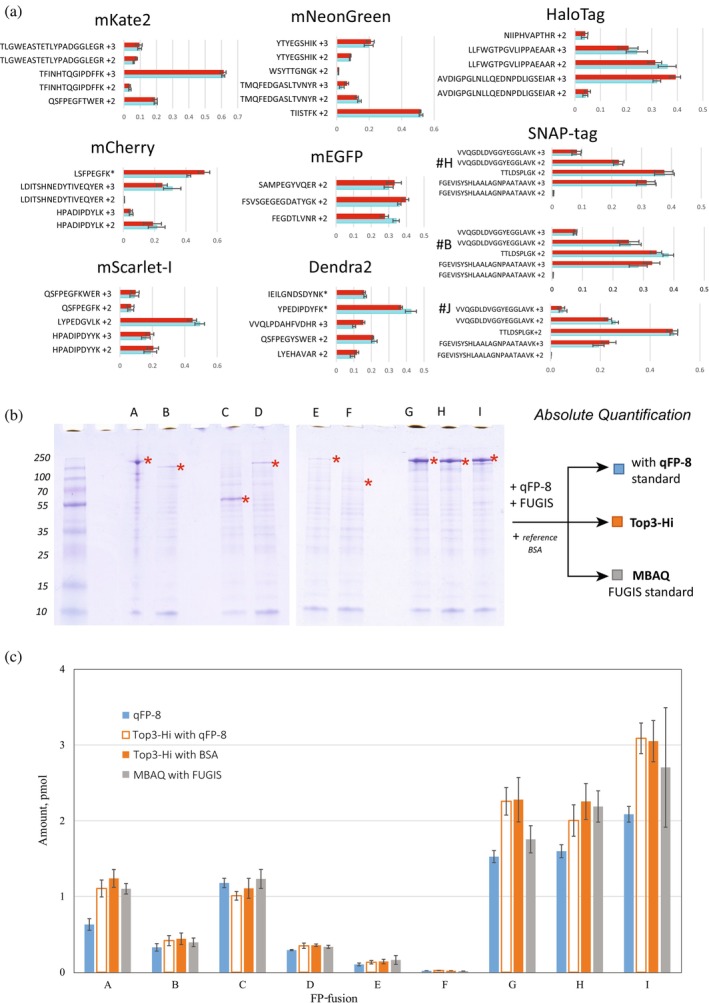
Validation of absolute quantification of FP‐fusions using qFP‐8 standard. (a) Ratios of normalized abundances (*x*‐axis) of quantotypic peptides (*y*‐axes) originating from qFP‐8 (in red) and from corresponding FP‐fusions (in turquoise). Different charge states of the same peptide are shown as separate bars. Asterisks (*) designate tryptic peptides with the added abundances of mis‐cleaved forms. #B, #H, and #J stand for FP‐fusions with Snap‐tag located at the C‐terminus, in the middle and the N‐terminus of the full‐length sequence, respectively (Table S2). (b) FP‐fusions #A to #I were analyzed by three methods: targeted quantification with qFP‐8 and untargeted quantification by Top3‐Hi (Silva et al. 2006) and Best 3 (MBAQ) (Raghuraman et al. 2022) methods. Bands corresponding to full‐length FP‐fusions are designated with an asterisk (*); bands of MW markers are at the left‐hand side. (c) Molar amounts of FP‐fusions per gel band determined by qFP‐8 standard (blue bars); by Top3‐Hi method using qFP‐8 (unfilled bars) and by BSA as references (orange bars); by MBAQ/FUGIS (gray bars). Error bars are SD (*n* = 4).

To address the first question, we co‐digested each of nine FP‐fusions containing different FPs (coded #A to #I in Table S1 and Data S1) with the isotopically labeled qFP‐8 standard. Then, for each fusion protein, we compared normalized ratios of intensities of XIC peaks of quantotypic peptides from its FP tag and from the qFP‐8 standard (Table S2) (Kumar et al. 2018; Rzagalinski et al. 2022). Excellent agreement between the ratios of 27 out of the total of 30 native (from FP‐fusions) and isotopically labeled (from qFP‐8) peptide pairs suggested complete digestion and unbiased peptide recovery, regardless of the FP tag location in the full‐length FP‐fusion sequence (Figure 2a).

Next, we demonstrated that the abundance of quantotypic peptides from the FP tag truly reflected the abundance of the full‐length FP‐fusion. To this end, each of nine FP‐fusions was co‐digested with qFP‐8 and with generic protein standard FUGIS designed for rapid proteome‐wide absolute quantification (Raghuraman et al. 2022), and analyzed the digests by LC–MS/MS. The molar amount of each FP‐fusion was independently determined in four ways (Figure 2b and Table S3): (a) by targeted quantification using qFP‐8 as described above (Figures 1b and S2); (b) by “best 3” peptides in the fusion digest whose abundance was referenced to median abundance of peptides from the FUGIS standard (MBAQ method, see Raghuraman et al. 2022); and (c) by “top 3” peptides in the fusion digest whose abundance was referenced to three most abundant peptides in the digest of full‐length qFP‐8; or (d) of BSA (Silva et al. 2006). Prior to the LC–MS/MS analysis we separated FP‐fusions by 1D SDS PAGE and digested corresponding protein bands in‐gel (Figures 2b, S3, and S4) to ensure that only full‐length proteins were analyzed. We underscore that, in contrast to the method a, the methods b–d did not rely on the identity of endogenous (from fusions) and standards (from FUGIS or qFP‐8) peptides. Best3 and top3 methods also use different principles of peptides selection. Consequently, peptides picked for “best 3” (method b) and “top 3” (methods c and d) quantification could differ even when selected from the same protein digest. We applied methods b–d to test if protein quantification that does not rely on peptides identity is concordant and can be used to benchmark the identity‐based quantification by qFP‐8.

Molar amounts of FP‐fusions were determined by the three independent methods with better than 20% precision (Figure 2c and Data S2). This was particularly encouraging since untargeted methods (MBAQ/FUGIS and “top 3”) were designed for rapid proteome‐wide quantitative profiling. For targeted quantification using qFP‐8, coefficients of variance (CVs) among peptide proxies of the same protein were, on average, 14%. The molar amount of the double‐tagged FP‐fusion #J independently determined using mEGFP and Snap‐Tag peptide proxies differed by only 15% (Table S4). We underscore that absolute quantification was performed in whole cell lysates (rather than stock solutions of purified protein standards) in which protein expression levels varied by more than 100‐fold for FP‐fusions (Table S4) and 400‐fold for FPs (Table S5). Depending on the rate of FPs expression (Table S5), we projected that FPs could be quantified in extracts of 10–100 HeLa cells (Figure S5) and has the potential to reach single‐cell level sensitivity using the method of PRM (Bourmaud et al. 2016) that compares the intensities of matching fragments in MS/MS spectra of native and labeled peptides.

We note that in the untargeted method b (“best 3”) and method c (“top 3 Hi”) peptides were mostly selected from a non‐FP part of FP‐fusions. Restricting the selection to sequences of FP tags biased the quantification (Data S2) due to their lower ionization capacity, as indicated by a below‐average intensity of their XIC peaks compared to other tryptic peptides. At the same time, targeted quantification with FP proxies allowed us to quantify fusions with proteins that yielded no suitable quantotypic peptides, such as FUS (FP‐fusion #B) or Claudin (FP‐fusion #C) (Data S1).

In summary, we concluded that the qFP‐8 standard is suitable for the targeted absolute quantification of fusions comprising any combination of FP and non‐FP sequences.

### 2.3 Peptides covering linear and cyclic forms of fluorescent chromophores

Fluorescent chromophore maturation influences FP‐fusion detection and quantification by spectroscopy. However, there is currently no generic assay to determine what fraction of the FP‐fusion is having the mature fluorescent chromophore (Chudakov et al. 2010; Evdokimov et al. 2006). “Dark” and fluorescent chromophores are structurally distinct (Chudakov et al. 2010; Craggs 2009; Subach et al. 2010; Subach and Verkhusha 2012) and we reasoned it should be possible to quantify both forms in LC–MS/MS experiment.

**FIGURE 3:**
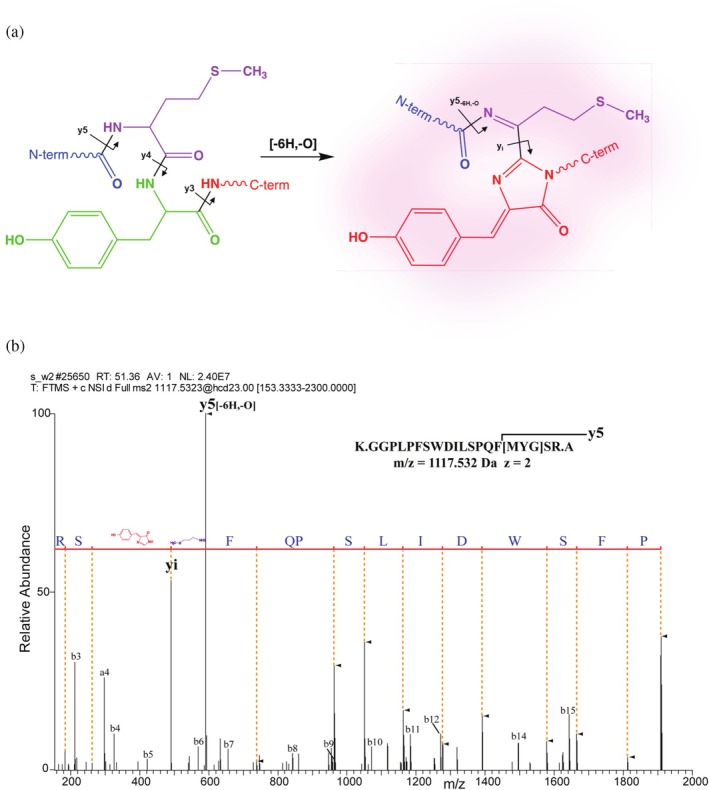
Chemical structure and MS/MS fragmentation of the red‐type chromophore containing peptide from mScarlet‐I fluorescent protein. (a) Chemical structures of “dark” (linear) and cyclic (fluorescent) chromophore …MYG… in the GGPLPFSWDILSPQMYGSR peptide (adapted from Miyawaki et al. 2012). Arrows indicate peptide bonds cleaved upon MS/MS fragmentation; *y*_*i*_ fragment is produced by cleavage of the cyclic chromophore adjacent to a 4‐(*p*‐hydroxybenzylidene)‐5‐imidazolinone moiety (in red). (b) MS/MS spectrum of GGPLPFSWDILSPQMYGSR peptide with the cyclic chromophore. *Y*‐ions comprising the chromophore (starting from *y*_5_) were detected with a mass shift of Δ*m* = −22.047 Da compared to corresponding fragments of the linear peptide and are designated with filled triangles. Fragments that do not contain the chromophore (*a*‐ and *b*‐ions and *y*_1_, *y*_2_‐ions) have *m*/*z* expected for the linear form of the peptide.

To this end, in LC–MS/MS analyses of FP digests (Table S1, proteins #1–6 and 9) we sought peptides that: (i) comprise the known chromophore sequence and (ii) whose masses differ by the equivalent loss of two hydrogen atoms and water (Δ*m* = −20.031 Da) or four hydrogen atoms and water (Δ*m* = −22.047 Da). These mass losses are characteristic of the mature cyclic forms of green‐ and red‐type chromophores (Figure 3a), respectively (Adam et al. 2009; Bravaya et al. 2012; Clavel et al. 2016; Pletnev et al. 2010; Subach and Verkhusha 2012). For all seven FPs, both linear and cyclic forms of chromophore‐containing peptides were detected in FT MS spectra and confirmed by MS/MS (Figures S6 and S7). Interestingly, MS/MS spectra of peptides with mature red‐type chromophore also contained the characteristic *y‐*ion produced by internal cleavage of its cyclic structure (designated “*y*_*i*_” in Figure 3; Figures S6 and S7b). No such cleavage was observed in peptides with green‐type chromophores or any intermediate form of the red chromophore.

As expected, in MS/MS spectra we observed the same mass shift Δ*m* = −20.031 Da for all *y*‐ions comprising cyclic green chromophore, as compared to its linear form. One notable exception was a peptide containing the …TYG… chromophore in EGFP. There chromophore‐containing *y*‐ions lost C_2_H_4_O moiety (Δ*m* = −44.026 Da), presumably from the N‐terminal threonine residue of the chromophore tripeptide (Figure S6b).

Importantly, the ratio of XIC peak intensities of cyclic and linear chromophore‐containing peptides corroborated the observed fluorescence. In the red FP mScarlet‐I (protein 2; Table S1), 72% of GGPLPFSWDILSPQMYGSR peptide (chromophore sequence underlined) contained cyclic chromophore. In contrast, 97% of this peptide in mScarlet‐I non‐fluorescent mutant M190 (Romero Romero et al. 2024) expressed under the same conditions included linear chromophore (Table S6). Slow maturing FP dsRed‐Express protein (protein 9; Table S1) contained less than 0.1% of the mature cyclic form (Table S6 and Figure S7), whereas ca. 70% and 29% of its chromophore occurred in the linear and intermediate forms, respectively.

We therefore concluded that the proportion of “dark” and fluorescent fractions together with the total molar abundance of the full‐length FP‐fusion can be determined within the same LC–MS/MS analysis and enable quantitative monitoring of FP maturation kinetics.

### 2.4 Absolute quantification of FP‐fusions to study protein expression in cell‐free systems

Cell‐free expression of FP‐fusions is an attractive system for studying the mechanism and kinetics of protein biogenesis (Gonzales et al. 2022; Hunt et al. 2025; Silverman et al. 2020; Tuckey et al. 2014) that takes advantage of facile and scalable readout by fluorescence spectroscopy. However, physicochemical and structural properties of FPs can compromise the correlation between measured fluorescence and actual concentration of their fusions (Geertsma et al. 2008). We demonstrate that combining fluorescence spectroscopy with absolute quantification of FP‐fusions by LC–MS/MS overcomes these analytical challenges. Furthermore, it enables the implementation of a sequential kinetic model to describe the interplay between transcription, translation, and folding.

**FIGURE 4:**
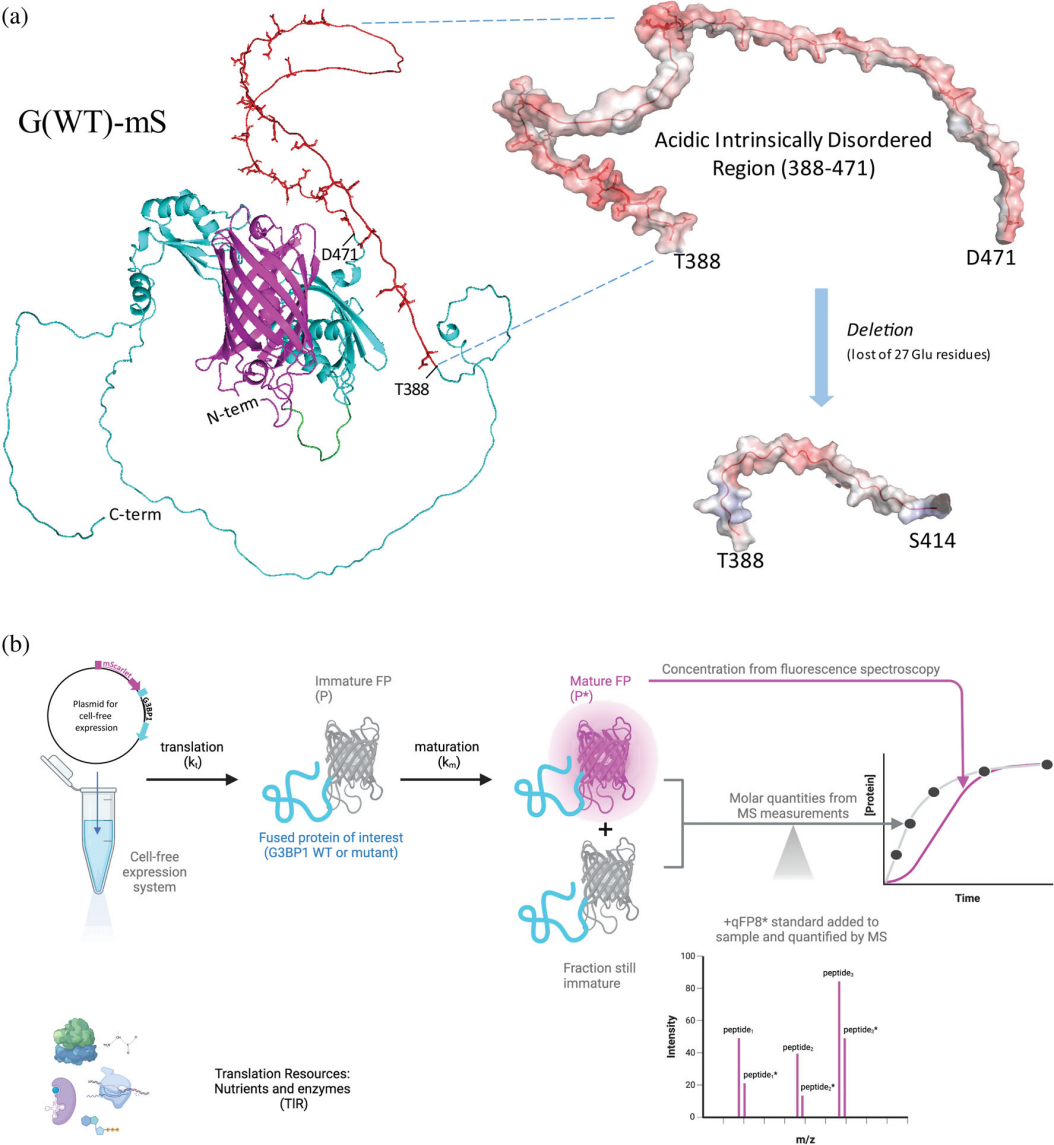
Studying the expression kinetics of G3BP1 and its mutant in cell‐free protein expression systems. (a) Predicted 3D structure of G3BP1 fused to mScarlet (G(WT)‐mS) and its intrinsically disordered region (IDR) in WT and IDR deletion mutant; mScarlet‐I is shown in pink, G3BP1 in magenta, with IDR (side chains of glutamic acid residues extended; red) and a short spacer sequence (green). The inset shows negative surface charge of the acidic IDR in G(WT)‐mS (388–471 aa) before and after removal of glutamic acid enriched sequence stretch (Guillen‐Boixet et al. 2020). (b) Workflow to study translation and maturation kinetics by LC–MS/MS and fluorescence spectroscopy.

As a test protein, we chose the human intrinsically disordered protein G3BP1 (Uniprot Q13283). It is a 52 kDa mRNA binding protein essential for liquid–liquid phase separation and formation of stress granules (Guillen‐Boixet et al. 2020; Yang et al. 2020). The glutamic acid‐rich intrinsically disordered region (IDR) of the G3BP1 sequence between amino acid residues 142–225 acts as a negative regulator, whose net charge determines the saturation concentration for phase separation with mRNA (Guillen‐Boixet et al. 2020). Using fluorescence spectroscopy and quantitative mass spectrometry we compared the yield and kinetics of expression of G3BP1 and its mutant, where glutamic acid residues were removed from the IDR (Guillen‐Boixet et al. 2020) changing its formal net charge at pH 7.4 from −17 to +11 (Figures 4a and S8). For this purpose, the red FP mScarlet‐I was fused to the N‐terminus of G3BP1 and of its mutant (these FP‐fusions are further termed G(WT)‐mS and G(mut)‐mS for the G3BP1 and the mutant, respectively). The corresponding vectors were expressed in *E. coli* based cell‐free expression system PURExpress (further referred to as PURE) and *S. frugiperda* cell extract TnT®T7 (further referred to as TnT) (Merk et al. 2015; Shimizu and Ueda 2010). At chosen time points the expression was quenched and the reaction mixture separated by 1D SDS PAGE. The Coomassie stained band matching the apparent molecular weight (*M*_*r*_) of the full‐length G(WT)‐mS or G(mut)‐mS proteins was excised, in‐gel digested and fusion proteins quantified by LC–MS/MS using mScarlet‐I peptide proxies from the q‐FP8 standard (Figures 1b and S2). In a parallel experiment, we used a plate reader to record the total fluorescence of the reaction mixture at the channel corresponding to mScarlet‐I fluorescence (Figure 4b). We chose to include a gel separation step prior LC–MS/MS to only compare the levels of full‐length expressed proteins. Partially expressed fragments with truncated C‐terminus could bias LC–MS/MS quantification (Rzagalinski et al. 2022), but in these experiments they did not contribute to the total fluorescence (Figure S9).

G(WT)‐mS and G(mut)‐mS proteins were quantified by LC–MS/MS using three mScarlet‐I peptide proxies in the qFP‐8 (Figure 1) with inter‐peptides CV of less than 15%. Their yield determined by mass spectrometry after 4 h of protein expression differed significantly between PURE and TnT systems. In PURE the concentration for both proteins exceeded 3 μM, whereas it was ca. 20‐fold lower in TnT (Data S3). Consistent with the previous reports that eukaryotic cell‐free systems could add post‐translational modifications to expressed proteins (Kinoshita‐Kikuta et al. 2021; Oza et al. 2015; Suzuki et al. 2006), we observed that G3BP1 expressed in the TnT system was phosphorylated at serine residues flanking the acidic IDR, as was also observed in living cells (Sahoo et al. 2018; Tourriere et al. 2001; Yang et al. 2020) (Figure S10 and Table S7). Since phosphorylation could promote G3BP1 dimerization (Guillen‐Boixet et al. 2020) and affect protein fluorescence (Pope et al. 2020), we chose the PURE system to study its expression kinetics.

**FIGURE 5:**
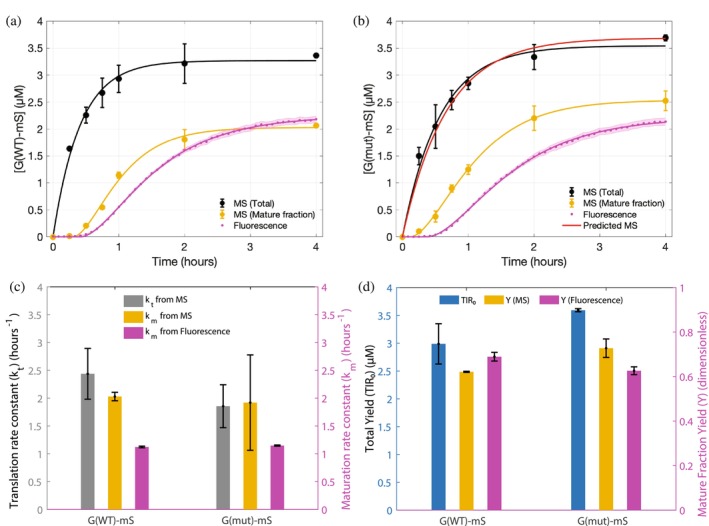
Kinetic traces, constants, and yield of the G3BP1 fusions expressed in PURE. Kinetic traces for mScarlet‐I fusions of G(WT)‐mS (panel a) and G(mut)‐mS (panel b) monitored by mass spectrometry (black) and fluorescence spectroscopy (magenta). Concentration of the fusion having a Scarlet cyclic chromophore determined by mass spectrometry is in amber. Total amount of expressed protein predicted by the kinetic model from fluorescence curve and single mass spectrometry measurement is in red (panel b). Translation and maturation rate constants (panel c) and overall yields (panel d) were calculated by fitting mass spectrometry and fluorescence measurements.

Kinetic curves of both G(WT)‐mS and G(mut)‐mS showed substantial (ca. 35%) difference between protein concentrations measured by mass spectrometry (black line) and by spectroscopy (magenta line) (Figure 5a,b and Data S3 and S4). A considerable fraction of the FP‐fusion failed to make the fluorescent chromophore even at the end time point (4 h) of expression and thus remained “dark.” In line with our observations, it has been reported that, depending on conditions, ca. 25–40% of various FPs (Dunsing et al. 2018) and ca. 14% of mScarlet‐I (Prangsma et al. 2020) remain non‐emissive. This prompted us to quantify the fraction of fusion protein having the mature cyclic chromophore in the mScarlet‐I tag by mass spectrometry (amber line) from the areas of XIC peaks of precursor ions of cyclic and linear forms of the chromophore‐containing peptide (Data S7 and Figures S6a and 3b).

Comparison of the amber and black lines indicated that chromophore maturation was significantly slower than protein translation. Notably, chromophore maturation (amber line) was faster than the increase in detected fluorescence (magenta line). However, by the end point, the entire protein fraction with the mature chromophore became fluorescent. Both the mutant and WT proteins were produced at similar molar concentrations and their expression was not considerably affected by the removal of the highly acidic IDR.

### 2.5 Kinetic model for protein expression and maturation of the fluorescent chromophore

Since mass spectrometry quantifies the total amount of fusion protein and fluorescence spectroscopy only quantifies its fraction having FP tag with mature chromophore, we constructed a first‐order sequential kinetic model (Gonzales et al. 2022; Macdonald et al. 2012; Marshall and Noireaux 2019) to estimate the rate constants of fusion translation and of chromophore maturation $k_{t}$ and $k_{m}$, respectively.

A cell‐free expression system has a finite amount of translation resources (TlR). Our model is based on the consideration that an initial translation resource ${\left[\mathrm{TlR}\right]}_{0}$ is fully converted into protein product, and comprises two consecutive events (Figure 4b): TlR is translated into the protein with immature chromophore P, a fraction of which becomes the final mature protein $P_{m}$. We further introduced a fractional yield (*Y*) for the conversion of P to $P_{m}$ because FP maturation can proceed through multiple branched pathways with competing chemical fates, rather than following a single obligate route (Strack et al. 2010).

$$\mathrm{TlR}\;\overset{k_{t}}{\to }P,$$

$$Y\times P\overset{k_{m}}{\to }P_{m}.$$

The differential equations associated with the model and their respective solution steps are presented in Data S7. From the analytical solutions the mass spectrometry data for total protein $\left[P_{T}\right]$ was fit to

(1) $$\left[P_{T}\right]={\left[\mathrm{TlR}\right]}_{0}\left(1-e^{-k_{t}.t}\right),$$

which plateaued at ${\left[\mathrm{TlR}\right]}_{0}$ at equilibrium (i.e., *t* = ∞).

The mature fraction as detected by mass spectrometry or by fluorescence spectroscopy was fit to the analytical solution of $\left[P_{m}\right]$,

(2) $$\begin{array}{l} \left[P_{m}\right]=(\frac{k_{t}.k_{m}.Y.{\left[\mathrm{TlR}\right]}_{0}}{k_{t}-k_{m}}\left(\frac{e^{-k_{t}.\left(t-t_{0}\right)}}{k_{t}}-\frac{e^{-k_{m}.\left(t-t_{0}\right)}}{k_{m}}\right)\\ \;+Y\;.\;{\left[\mathrm{TlR}\right]}_{0}).\;H(t-t_{0}), \end{array}$$

which plateaued at $Y\times {\left[\mathrm{TlR}\right]}_{0}$ at equilibrium (i.e., *t* = ∞).

For the protein fraction with mature chromophore obtained by mass spectrometry or by fluorescence spectroscopy, a Heaviside step function *H* was added to fit the data from a time point $t_{0}$ from when the signal began to grow to account for hidden processes preceding maturation (Figure 5a,b). Having obtained the protein translation rate constant $k_{t}$ by fitting the mass spectrometry curve with Equation (1) it was then used as a known parameter in Equation (2). The Equation (2) was subsequently used to fit the mature fraction data from mass spectrometry or from the fluorescence spectroscopy to obtain two respective maturation rate constants $k_{m}$ (Figure 5c).

From the fits for both WT and mutant fusions, the protein translation rate constant $k_{t}$ calculated for G(WT)‐mS was 2.44 ± 0.46 h^−1^ and its maturation rate constant $k_{m}$ obtained from fluorescence spectroscopy was 1.12 ± 0.01 h^−1^, which corresponded to a maturation half‐time of 37.1 ± 0.4 min (Data S5). This value is close to, on average, 31.4 ± 2.5 min reported for the mScarlet‐I chromophore, as averaged from the values reported by Balleza et al. (2018), McCullock et al. (2020), and Guerra et al. (2022). We speculate that this less than 20% difference in maturation half‐times could reflect the impact of unusual highly acidic sequence in G(WT)‐mS.

Next, we wanted to test the predictive potential of our model. We asked whether we could simulate a total protein curve for a fusion using previously published maturation half‐time estimates. To this end, we took experimentally obtained fluorescence curves and a single data point from mass spectrometry at the 4 h mark for the G(mut)‐mS. The procedure was applied in the reverse order: we used the mScarlet‐I maturation half‐time value of 31.4 ± 2.5 min (see above), fit the fluorescence data for the G(mut)‐mS and calculated a theoretical translation rate constant ($k_{t}$). The obtained theoretical $k_{t}$ of 1.86 ± 0.38 h^−1^ together with the single mass spectrometric 4th hour data point as [TlR]_0_ were subsequently used to predict a total protein curve for G(mut)‐mS, which agreed very well with the curve obtained by mass spectrometry (Figure 5b).

Notably, the maturation rate constants for both fusions calculated using mass spectrometry data for mature chromophore fraction (amber curves) were higher than calculated from fluorescence measurements (Figure 5c). The corresponding maturation half‐times (16.5 ± 0.28 and 21.7 ± 9.59 min for G(WT)‐mS and G(mut)‐mS, respectively) were less than the values from the literature (Balleza et al. 2018; Guerra et al. 2022; McCullock et al. 2020). This suggested that there may be an additional step preceding the formation of the fluorescent fraction of the fusions. To test this assumption, we constructed an extended model (details are in Data S7) according to the schematic,

$$\mathrm{TlR}\overset{k_{t1}}{\to }P_{0}\overset{k_{t2}}{\to }P\overset{k_{c}}{\to }P_{c}\overset{k_{f}}{\to }P_{f},$$

where the total nutrient resource TlR is converted into a precursor, $P_{0}$, which accounts for the delay in the fluorescence and mature fraction curves. Then it proceeds to the immature fraction *P*, as in the original model, and then to a fraction $P_{c}$ having “dark” chromophore, which is then converted to the final fluorescent form via a possible folding step, $P_{f}$.

Introducing an intermediate “dark” state, $P_{c}$ is necessary because FPs maturation is a multi‐step process involving multiple non‐fluorescent intermediates (Barondeau et al. 2003; Reid and Flynn 1997; Stepanenko et al. 2011) which are particularly pronounced for red FPs (Verkhusha et al. 2004). For the purpose of kinetic modeling, these intermediates could be lumped into a single “dark” $P_{c}$ state in which the chromophore could be detected by mass spectrometry, but is not emissive. Hereon, a fractional yield (*Y*) of the $P_{c}$ state branches off to form an emissive chromophore.

Numerical fits of the same data to the corresponding differential equations for this extended model are in Data S7, Figure S11, and Data S5. From the resulting rate constants, an effective rate constant was derived using the formalism,

$$k_{\mathrm{eff}}={\left({k_{c}}^{-1}+{k_{f}}^{-1}\right)}^{-1},$$

which combines the chromophore maturation and protein folding steps. The effective rate constant was then compared to the respective maturation rate constant $k_{m}$ from the original model. The percentage difference between $k_{\mathrm{eff}}$ and $k_{m}$ was below 10% for both G(WT)‐mS and G(mut)‐mS, supporting the hypothesis of the sequential maturation process through an intermediate state with a correctly assembled chromophore, but lacking fluorescence (Figure S11c).

Overall, the data showed that WT and mutant forms of the intrinsically disordered protein G3BP1 have very close yields of expression (differing by less than 15%) and comparable translation rates (Figure 5c,d). This additionally confirms that the change in net charge does not have a significant impact on the expression yield and kinetics of the protein. The slightly increased yield of G(mut)‐mS might be attributed to its shorter length.

Taken together, our model allows the determination of maturation rate constants for FP‐reporters in cell‐free protein expression systems. It could predict protein expression curves based on a facile fluorescence readout combined with a single protein quantification experiment by mass spectrometry. The model can be instrumental for high throughput screens aiming at or relying upon expression of isoforms, mutants, or homologous proteins tagged with the same FP.

However, it does not aim to explain the detailed molecular mechanism underlying the individual steps of protein expression and chromophore biogenesis. Rather, it was constructed as a coarse‐grained framework to quantify the kinetics of expression and chromophore maturation in FP‐fusions using three experimental observables: the total protein concentration, its fraction containing the mature chromophore and the fluorescent fraction. Effectively, the model relies on three major assumptions. First, the cell‐free reaction is treated as a closed resource‐limited system with finite translational capacity (TlR). Second, we adopted a linear sequential pathway, even though branched maturation schemes have been reported (Strack et al. 2010). Third, the fitted parameters are interpreted as effective (lumped) rate constants. Finally, we included a simple phenomenological delay to account for upstream initiation, transcriptional and early translation processes that are not directly observed in the present measurements.

With these assumptions applied, the mass‐action based reactant‐intermediate(s)‐product type sequential model provides a generalizable way that can be used to fit any experimental data that follows a similar reaction scheme with an intermediate (here, the immature FP‐reporter). Although over the course of the reaction the intermediate state is generally invisible, the model allows us to infer and track its dynamics (Figure S12).

## 3 CONCLUSIONS

We have designed and validated a simple and robust analytical approach for determining the molar abundance of fluorescent protein fusions. It relies on the highly expressed isotopically labeled chimeric protein qFP‐8 that enables absolute quantification of FP‐fusions with two self‐labelling tags as well as with more than 615 proteins from six major FP families sharing with qFP‐8 at least one quantotypic peptide (Data S6). There is no need to synthesize individual peptide standards or use purified FPs as calibrants and the protocol can be applied to any FP‐fusion with no further adjustments. Also, purification of qFP‐8 chimeric standard is not required. The same analysis quantifies the molar fraction of mature cyclic chromophore, which is directly relevant to visible fluorescence.

We envision that this technically straightforward proteomics method will support diverse applications, including quantifying expressed FP‐fusions and assessing their maturation rate in cells and tissues or using FP‐fusions as quantitative reporters, among others. However, an apparent limitation of the method is that, in contrast to quantitative fluorescence microscopy, it loses spatial information regarding the localization of the corresponding FP‐fusion. While, in principle, sensitivity at the single cell level may be achievable, there are considerable technical challenges associated with the unbiased recovery of proteins material from individual cells and its preparation for LC–MS/MS analysis.

We further combined mass spectrometry‐based quantification with fluorescence spectroscopy to monitor the kinetics of fusions expression in cell‐free systems. Effectively, we were able to delineate the kinetics of protein translation, chromophore maturation and formation of genuine fluorescent protein and then integrate these multifaceted kinetic data into a mathematic model. Together with computational prediction of protein structures, this could advance our understanding of the interplay between protein sequence, translation, folding and structure. Finally, we envision that FP‐fusions quantification at the single cell level will expand the analytical scope of system and synthetic biology by aligning the spatial precision of fluorescent microscopy with exact molar quantities of imaged proteins. It is also intriguing to explore if FP‐fusions with precisely known molar quantities could serve as a generic internal standard (Raghuraman et al. 2022) to determine the molar abundance of endogenous proteins detected in the same microscopy experiment and contribute to understanding the molecular composition of labile protein condensates (McCall et al. 2025).

## 4 MATERIALS AND METHODS

Chemicals and key resources are in Data S7.

### 4.1 Preparation of chimeric protein standards, recombinant FPs and FP‐fusions

FPs were expressed in *E. coli*, FP‐fusions—in insect cells (*Trichoplusia ni*); qFP‐8 was metabolically labeled as described in Kumar et al. (2018) and Raghuraman et al. (2022). Cells were lysed, aliquots of whole lysates separated by 1D SDS PAGE and visualized by Coomassie staining. The positions of full‐length FP‐fusions on the gel were determined by fluorescence gel imaging and by Western blotting using a primary antibody directed against the HRV 3C‐site. For quantification of FP‐fusions in method validation experiment, gel bands corresponding to the fusion, standard chimeric proteins qFP‐8 and FUGIS, and 1 pmol of the reference protein BSA were co‐digested *in‐gel* with trypsin. For detection of chromophore‐covering peptides, depending on the FP sequence, corresponding FP bands were *in‐gel* digested with trypsin or Asp‐N protease. For FP quantification in stably transfected cells, HeLa and HCT116 cells were counted prior lysis and then processed as above. Peptides were extracted, dried down, and subjected to LC–MS/MS analysis.

Details of the sample preparation are in Data S7.

### 4.2 Protein expression kinetics study in cell‐free systems

Plasmids coding for G3BP1 and the mutant N‐terminally fused with mScarlet‐I were designed and optimized for insect and bacterial expression systems as described in Data S7. The plasmids were then used in PURExpress® and TnT® T7 cell‐free Protein Expression Systems according to the manufacturer protocol. For absolute quantification by mass spectrometry, 10 μL aliquots were withdrawn with 15–30 min interval from the master solution, quenched with 10 μL Laemmli buffer and separated by SDS PAGE. Gel regions at around 80 and 73 kDa corresponding to G(WT)‐mS and G(mut)‐mS were *in‐gel* digested with trypsin; qFP‐8 standard and reference BSA were separately co‐digested *in‐gel* and aliquots spiked into digests of the model proteins prior LC–MS/MS analysis.

In parallel, the expression of mScarlet tagged G3BP1 variants was tracked on a TECAN Spark 20M plate reading fluorimeter. After initiation of expression, fluorescent signal was measured during 4 h at 569/594 nm excitation/emission wavelength. Obtained arbitrary fluorescence units were converted to protein concentration using calibration curves built with purified mScarlet‐I protein.

### 4.3 Mass spectrometry analysis and absolute quantification

Peptide mixtures were analyzed by LC–MS/MS on a nanoUPLC Ultimate 3000 interfaced to a Q Exactive HF hybrid mass spectrometer; MS3 experiment was carried on a LTQ Orbitrap Velos mass spectrometer (all from Thermo Fisher Scientific, Bremen). Data were acquired in DDA mode and in DDA with inclusion list of precursor *m*/*z* (see Data S7). To avoid carryover, two blank runs were performed after each sample analysis.

Spectra were processed with FragPipe software suit v.17.1 (Kong et al. 2017) or matched by Mascot software (v.2.2.04, Matrix Science). Intensities of native and isotopically labeled peptide precursors were extracted from FragPipe output by in‐house scripts. Absolute quantification using isotopically labeled chimeric standard qFP‐8 and FUGIS was performed as described in Kumar et al. (2018) and Raghuraman et al. (2022).

Modeling procedures are described in Data S7.

## AUTHOR CONTRIBUTIONS

**Anna Shevchenko:** Conceptualization; investigation; writing – original draft; methodology; writing – review and editing. **Archishman Ghosh:** Conceptualization; investigation; writing – original draft; formal analysis; methodology; software; writing – review and editing. **Andrea Schuhmann:** Investigation; validation. **Aliona Bogdanova:** Resources; methodology. **Henrik Thomas:** Investigation; validation. **Viditha Rao:** Investigation; validation. **Eric R. Geertsma:** Resources; writing – original draft; methodology. **T.‐Y. Dora Tang:** Conceptualization; supervision; writing – original draft; funding acquisition; writing – review and editing. **Andrej Shevchenko:** Conceptualization; supervision; project administration; writing – original draft; funding acquisition; writing – review and editing; methodology.

## CONFLICT OF INTEREST STATEMENT

The authors declare no conflicts of interest.

## Supporting information


**Data S1.** List of FP‐fusions.
**Data S2**. Absolute quantities of FP‐fusions obtained by ms‐based Top3‐Hi and MBAQ methods.
**Data S3**. Concentration of the model FP‐fusions in PURExpress cell‐free expression system obtained by mass spectrometry.
**Data S4**. Concentration of the model FP‐fusions in PURExpress cell‐free expression system obtained by fluorescent spectroscopy.
**Data S5**. Kinetic model parameters.
**Data S6**. List of fluorescent proteins quantifiable with qFP‐8 protein standard.

**Data S7.** Extended description of reagents and experimental procedures.

**Figure S1.** Characterization of the chimeric protein standard qFP‐8 by LC–MS.
**Figure S2.** Workflow for absolute quantification of FPs and FP‐fusions with qFP‐8 chimeric protein standard.
**Figure S3.** Characterization of FP‐fusions ##A‐I by Western blot and fluorescent gel imaging.
**Figure S4.** Peptide mapping of FP‐fusions by mass spectrometry.
**Figure S5.** Absolute quantification of EGFP in stably transfected HeLa cells with qFP‐8 standard.
**Figure S6.** Fragmentation spectra of chromophore‐containing peptides from red‐ and green‐type FPs.
**Figure S7.** Chromophore‐containing peptides detected in red‐type FP dsRed‐express by mass spectrometry.
**Figure S8.** Removal of glutamic acid residues from intrinsically disordered region changed formal net charge of the mScarlet‐tagged protein G3BP1.
**Figure S9.** Analysis of short products of expression.
**Figure S10.** Detection of serine phosphorylation in G(WT)‐mS expressed in TnT cell‐free system by mass spectrometry.
**Figure S11.** Extended model.
**Figure S12.** Simulations of hidden variables using the original and extended models.
**Table S1.** List of fluorescent proteins, self‐labelling tags and their fusions.
**Table S2.** Peptide proxies included in qFP‐8 chimeric standard protein.
**Table S3.** MS‐based approaches for absolute quantification of proteins using peptide references and spiked protein standards.
**Table S4.** Amount of FPs‐fusions ##A‐J quantified using peptide proxies of the qFP‐8 chimeric standard.
**Table S5.** Examples of FP amounts in stably transfected cells quantified using qFP‐8 standard.
**Table S6.** Abundance of chromophore‐containing peptides in red FP mScarlet and dsRed‐express.
**Table S7.** Phosphorylation status of the G3BP1 peptide SSSPAPADIAQTVQEDLR detected by mass spectrometry in G(WT)‐mS and G(mut)‐mS expressed in TnT and PURE cell‐free expression systems.

## Data Availability

Source files with LC–MS/MS spectra in *.raw format are available at Edmond repository (Guerra et al. 2022; URL: https://doi.org/10.17617/3.UXWVQE). Scripts for the calculating kinetic parameters of expression and chromophore maturation of FP‐fusions are available at Edmond repository (URL: https://doi.org/10.17617/3.VGVZTS).
