# Supplementary material for: Absolute quantification of fluorescent protein fusions by mass spectrometry
Source: Protein Sci. 2026 Apr 6;35(5):e70556. doi: 10.1002/pro.70556 (PMC13051905; doi:10.1002/pro.70556)
Supplement: Supplementary file 3 — Figure S1. Characterization of the chimeric protein standard qFP‐8 by LC–MS. Figure S2. Workflow for absolute quantification of FPs and FP‐fusions with qFP‐8 chimeric protein standard. Figure S3. Characterization of FP‐fusions ##A‐I by Western blot and fluorescent gel imaging. Figure S4. Peptide mapping of FP‐fusions by mass spectrometry. Figure S5. Absolute quantification of EGFP in stably transfected HeLa cells with qFP‐8 standard. Figure S6. Fragmentation spectra of chromophore‐containing peptides from red‐ and green‐type FPs. Figure S7. Chromophore‐containing peptides detected in red‐type FP dsRed‐express by mass spectrometry. Figure S8. Removal of glutamic acid residues from intrinsically disordered region changed formal net charge of the mScarlet‐tagged protein G3BP1. Figure S9. Analysis of short products of expression. Figure S10. Detection of serine phosphorylation in G(WT)‐mS expressed in TnT cell‐free system by mass spectrometry. Figure S11. Extended model. Figure S12. Simulations of hidden variables using the original and extended models. Table S1. List of fluorescent proteins, self‐labelling tags and their fusions. Table S2. Peptide proxies included in qFP‐8 chimeric standard protein. Table S3. MS‐based approaches for absolute quantification of proteins using peptide references and spiked protein standards. Table S4. Amount of FPs‐fusions ##A‐J quantified using peptide proxies of the qFP‐8 chimeric standard. Table S5. Examples of FP amounts in stably transfected cells quantified using qFP‐8 standard. Table S6. Abundance of chromophore‐containing peptides in red FP mScarlet and dsRed‐express. Table S7. Phosphorylation status of the G3BP1 peptide SSSPAPADIAQTVQEDLR detected by mass spectrometry in G(WT)‐mS and G(mut)‐mS expressed in TnT and PURE cell‐free expression systems. [file PRO-35-e70556-s001.pdf]

List of Supplementary Figures and Tables

Figure S1. Characterization of the chimeric protein standard qFP-8 by LC-MS

Figure S2. Workflow for absolute quantification of FPs and FP-fusions with qFP-8 chimeric protein standard

Figure S3. Characterization of FP-fusions ##A-I by Western Blot and Fluorescent Gel Imaging

Figure S4. Peptide mapping of FP-fusions by mass spectrometry

Figure S5. Absolute quantification of EGFP in stably transfected HeLa cells with qFP-8 standard

Figure S6. Fragmentation spectra of chromophore-containing peptides from red- and green-type FPs

Figure S7. Chromophore-containing peptides detected in red-type FP dsRed-express by mass spectrometry

Figure S8. Removal of glutamic acid residues from intrinsically disordered region changed formal net charge of the mScarlet-tagged protein G3BP1

Figure S9. Analysis of short products of expression

Figure S10. Detection of serine phosphorylation in G(WT)-mS expressed in TnT cell-free system by mass spectrometry

Figure S11. Extended model

Figure S12. Simulations of hidden variables using the original and extended models

Table S1. List of Fluorescent Proteins, Self-labelling Tags and their Fusions

Table S2. Peptide proxies included in qFP-8 chimeric standard protein

Table S3. MS-based approaches for absolute quantification of proteins using peptide references and spiked protein standards

Table S4. Amount of FPs-fusions ##A-J quantified using peptide proxies of the qFP-8 chimeric standard

Table S5. Examples of FP amounts in stably transfected cells quantified using qFP-8 standard

Table S6. Abundance of chromophore-containing peptides in red FP mScarlet and dsRed-express

Table S7. Phosphorylation status of the G3BP1 peptide SSSPAPADIAQTVQEDLR detected by mass spectrometry in G(WT)-mS and G(mut)-mS expressed in TnT and PURE cell free expression systems

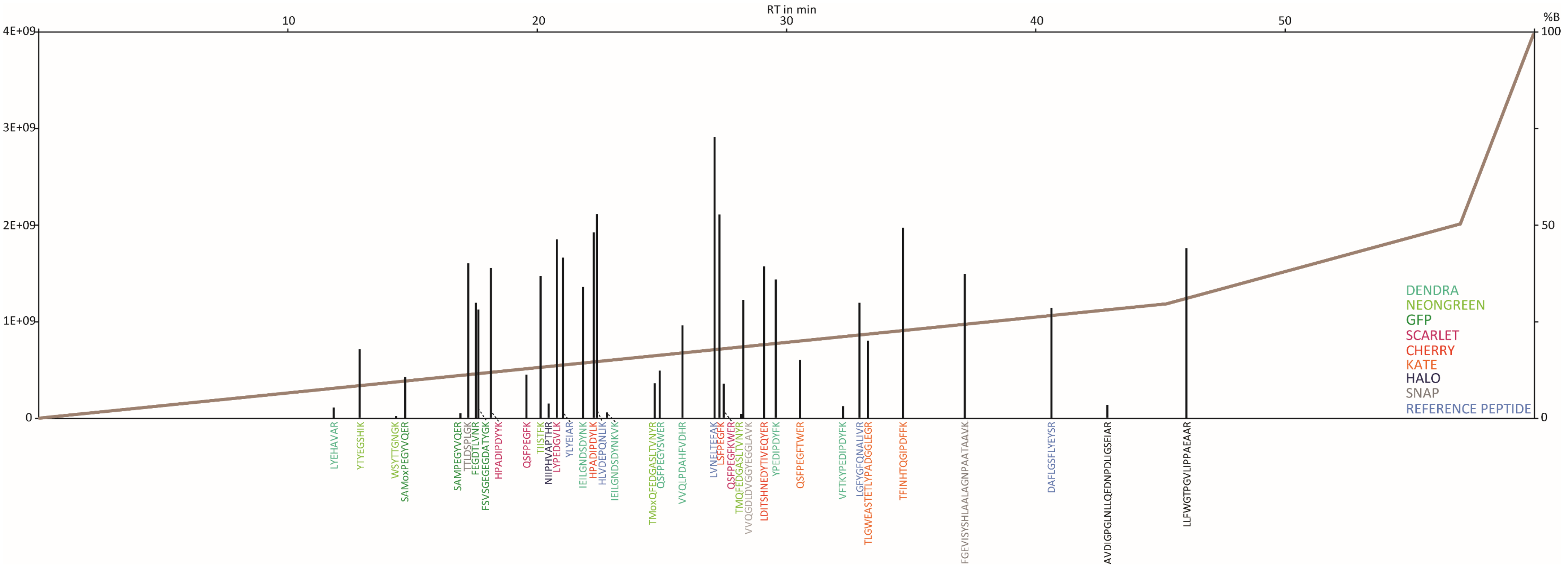

Supplementary Figure S1. Characterization of the chimeric protein standard qFP-8 by LC-MS.

150fmol of tryptic digest of the qFP-8 was injected. FP proxy peptides are colour-coded (see legend on the right-hand side). Vertical axis at the left-hand side shows the raw intensity of the peptide signal. The intensities of the +2/+3 charge states of the same peptide are summed. The intensity of Met-containing peptides is the sum of intensities native and mono-oxidized forms. The vertical axis at the right hand side shows % of acetonitrile (solvent B) in the chromatographic gradient; the gradient profile is shown as a continuous black line. The top horizontal axis shows the retention time.

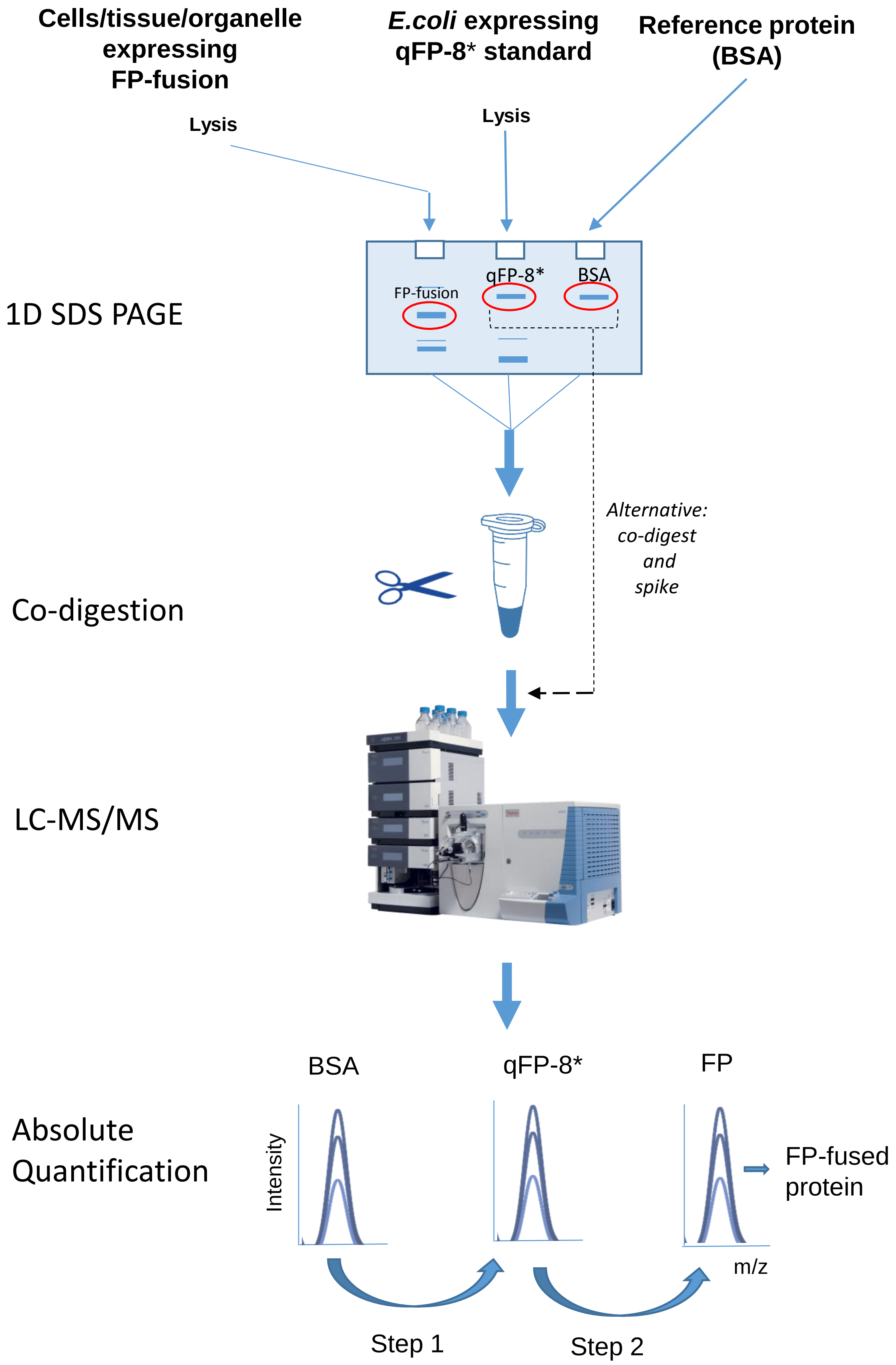

Supplementary Figure S2. Workflow for absolute quantification of FPs and FP-fusions with qFP-8 chimeric protein standard. "*" stays for $^{13}C_6{}^{15}N_4$-Arg and $^{13}C_6$ – Lys in metabolically labelled proteins. Dashed arrow: alternative sample preparation step in which qFP-8 and BSA are digested separately and spiked into the protein digest before LC-MS/MS analysis. This helps in scaling up the experiments.

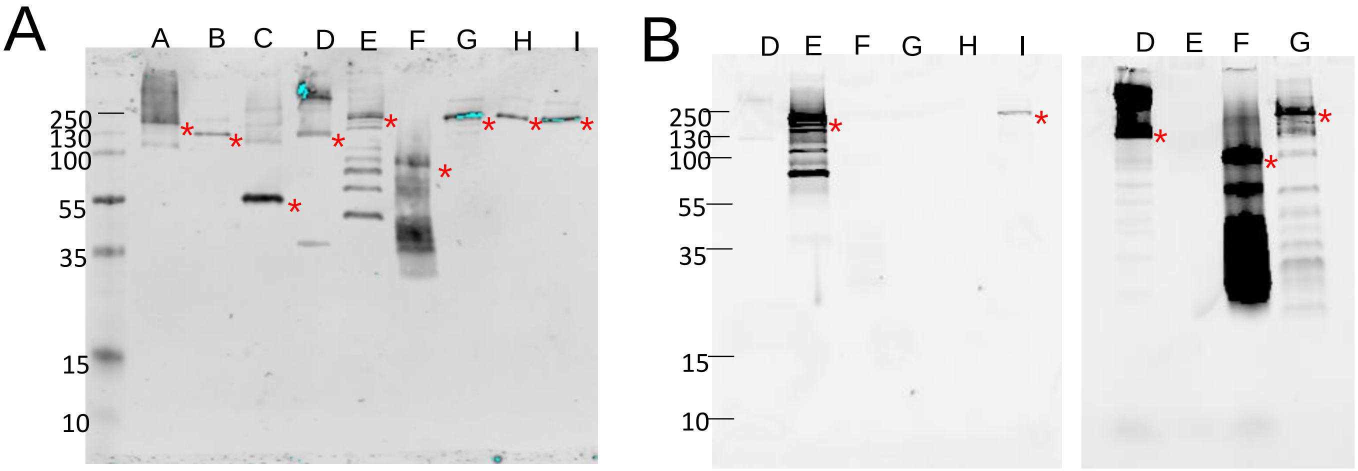

Supplementary Figure S3. Characterization of FP-fusions ##A-I by Western Blot and Fluorescent Gel Imaging.

Aliquots of lysates of insect cells expressing fusions ##A-I were separated by SDS PAGE; fluorescent gel imaging and Western blotting were performed using the same gel. #A-#I stays for the FP-fusion (Suppl. Dataset S1); bands corresponding to the position of full-length FP-fusions are designated with asterisk (*); molecular weight markers shown on the left. **A**: Western blot performed against 3C-cleavage site; **B:** fluorescent gel imaging acquired on Cy3 (left) and Cy5 (right) channels as described in Materials and Methods. Fusion A (fused FP – far-red mKate2) – fluorescence cannot be measured with available wave lengths.

Additional bands originate from incomplete translation and multimerization under non-reducing conditions. Intensive fluorescence and Western blot signals detected for fusion F below its full length sequence position indicated that full-length protein is only a minor fraction in the sample. Dendra-containing C-terminal fragments below 60kDa are x9 more abundant (quantified 160fmol) then its correctly expressed fusion (quantified 160 fmol and 19 fmol respectively). The case of fusion #F underlines the importance of gel separation to remove products of incomplete expression products before quantitative analysis of labelled proteins. In-solution analysis of fluorescence could bias the amount of fusion #F.

Fusion #A

1 MGSAWSHPQF EKGGGSGGGS GGSAWSHPQF EKLEVLFQGP AAAMDSSSGS
51 QGNGSFMDQN SLGILNMDNL KVFGDLSSFS KSGRRSGTGR DSPAVHERLR
101 EERLRALETL EKPRPSRSSQ AKSTALQMRL STNISELVTD DTIDLNSSSS
151 FALVTPATKG TNISFEPAEI TGRSTLCQAG KQRRREKPSL SVAEILKSSF
201 VEKARLLERK LQDASQAEAS YHLSRGSSSS LSDSFNCSRS LPRTADMSEL
251 SLNGGGGFSF GSASLAAEGQ DESFAPAELM QSKLVLGEIS WAQEFTAMPT
301 ATLSTQALKK IAAPTSEIET SVSNSVLAGD PDFSLGNYFQ TRSENIWNIV
351 SNSSPNRSRT NQPLLEPESN VTLDSVGEKT PQPDNKTYTK TDAITGSNLG
401 RNLMRKMQQD RIETALKSRN GLAAKETKRP PSSSEILSLS AIDKALRDID
451 LNSDTSTVEV VNHLWEHGRG NNYDDGENKE NQSSNSHAER TSCGSNKLTD
501 TMSFTDSVLN STDFRHLQQS ISRKPLSPLA DHPQITISRA DTDPVETEAE
551 ADIDEWPSTP VKEPSRRVRR TKISPRAASP SSSDGVRPLT CTEDENDEED
601 EDKTPVNKKR VSIATMGLIP RKASSLSSTR LDGCDVAVAS STERDFGISP
651 NLAKNLSPLS SPRSCLSSPL LDSTTSSDRR QLMSGAARKA NSSPAGSEAS
701 STSGFTASGR RGLGTSSVAV PRSISRCSNS SEFSHRDGKL LPLKVTHTTL
751 CWGSTKLRTD VRKSMQVKNT ADKRLVIRLG IQGPGFQLVG TDSSTITLQA
801 MECRSVVINF CPTVCGAAIG ALSFYAPPGA HNSNQPGLEI PLYGYGGSAS
851 ITINGLLKGP VGASFLTIGD VCELSAGPLS ANLKFHNKGP LTAFVGISVD
901 STVLMKLRLS EAFEVRPSRL ILPPNSERCV QILFRPNRED LKNILKKTAQ
951 VLTLANMRIF CGDEPNRQRM RSLVQRMSSR QREKLTSPML DSIWGEFPDE
1001 QPVRNIGQLN EPPEFILDLV TVVRIIDVAL TLNRDFDESS ESSLMFLPEA
1051 DETVLFRTIC VANSPTPTQS HLLEPVDELL EADTTTLTPV EQNWCVQPGL
1101 LEFGVGCSPL ATLSVTNSFC SRQLIEVTCN VPDLVRISPS ECYVSPDGGH
1151 AEIDVRLLRS PRQDLLEREP LIVVSMENER ISVPISFKFG APGSAGSAAG
1201 SGMVSELIKE NMHMKLYMEG TVNNHHFKCT SEGEGKPYEG TQTMRIKAVE
1251 GGPLPFAFDI LATSFMYGSK TFINHTQGIP DFFKQSFPEG FTWERVTTYE
1301 DGGVLTATQD TSLQDGCLIY NVKIRGVNFP SNGPVMQKKT LGWEASTETL
1351 YPADGGLEGR ADMALKLVGG GHLICNLKTT YRSKKPAKNL KMPGVYYVDR
1401 RLERIKEADK ETYVEQHEVA VARYCDLPSK LGHRLEVLFQ GPGSSHHHHH
1451 HSG

Fusion #B

1 MGSSHHHHHH SSGRMKIEEG KLVIWINGDK GYNGLAEVGK KFEKDTGIKV
51 TVEHPDKLEE KFPQVAATGD GPDIIFWAHD RFGGYAQSGL LAEITPDKAF
101 QDKLYPFTWD AVRYNGKLIA YPIAVEALSL IYNKDLLPNP PKTWEEIPAL
151 DKELKAKGKS ALMFNLQEPY FTWPLIAADG GYAFKYENGK YDIKDVGVDN
201 AGAKAGLTFL VDLIKNKHMN ADTDYSIAEA AFNKGETAMT INGPWAWSNI
251 DTSKVNYGVT VLPTFKGQPS KPFVGVLSAG INAASPNKEL AKEFLENYLL
301 TDEGLEAVNK DKPLGAVALK SYEEELVKDP RIAATMENAQ KGEIMPNIPQ
351 MSAFWYAVRT AVINAASGRQ TVDEALKDAQ TNSSSNNNNN NNNNNSSGRL
401 EVLFQGPAAA MASNDYTQQA TQSYGAYPTQ PGQGYSQQSS QPYGQQSYSG
451 YSQSTDTSGY GQSSYSSYGQ SQNTGYGTQS TPQGYGSTGG YGSSQSSQSS
501 YGQQSSYPGY GQQPAPSSTS GSYGSSSQSS SYGQPQSGSY SQQPSYGGQQ
551 QSYGQQQSYN PPQGYGQQNQ YNSSSGGGGG GGGGGNYGQD QSSMSSGGGS
601 GGGYGNQDQS GGGGSGGYGQ QDRGGRGRGG SGGGGGGGGG GYNRSSGGYE
651 PRGCGGGRGG RGGMGGSDRG GFNKFGGPRD QGSRHDSEQD NSDNNTIFVQ
701 GLGENVTIES VADYFKQIGI IKTNKKTGQP MINLYTDRET GKLKGEATVS
751 FDDPPSAKAA IDWFDGKEFS GNPIKVSFAT RRADFNRGGG NGRGGRGRGG
801 PMGRGGYGGG GSGGGGRGGF PSGGGGGGGQ QRAGDWKCPN PTCENMNFSW
851 RNECNQCKAP KPDGPGGGPG GSHMGGNYGD DRRGGRGGYD RGGYRGRGGD
901 RGGFRGGRGG GDRGGFGPGK MDSRGEHRQD RRERPYGAPG SSSGRENLYF
951 QGMDKDCEMK RTTLDSPLGK LELSGCEQGL HRIIFLGKGT SAADAVEVPA
1001 PAAVLGGPEP LMQATAWLNA YFHQPEAIEE FPVPALHHPV FQQESFTRQV
1051 LWKLLKVVKF GEVISYSHLA ALAGNPAATA AVKTALSGNP VPILIPCHRV
1101 VQGDLDVGGY EGGLAVKEWL LAHEGHRLGK PGLG

Fusion #C

1 MGSSHHHHHH SSGRLEVLFQ GPMAEIGTGF PFDPHYVEVL GERMHYVDVG
51 PRDGTPVLFL HGNPTSSYVW RNIIPHVAPT HRCIAPDLIG MGKSDKPDLG
101 YFFDDHVRFM DAFIEALGLE EVVLVIHDWG SALGFHWAKR NPERVKGIAF
151 MEFIRPIPTW DEWPEFARET FQAFRTTDVG RKLIIDQNVF IEGTLPMGVV
201 RPLTEVEMDH YREPFLNPVD REPLWRFPNE LPIAGEPANI VALVEEYMDW
251 LHQSPVPKLL FWGTPGVLIP PAEAARLAKS LPNCKAVDIG PGLNLLQEDN
301 PDLIGSEIAR WLSTLEISGG SAGSAAGSGA AAMASLGLQL VGYILGLLGL
351 LGTLVAMLLP SWKTSSYVGA SIVTAVGFSK GLWMECATHS TGITQCDIYS
401 TLLGLPADIQ AAQAMMVTSS AISSLACIIS VVGMRCTVFC QESRAKDRVA
451 VAGGVFFILG GLLGFIPVAW NLHGILRDFY SPLVPDSMKF EIGEALYLGI
501 ISSLFSLIAG IILCFSCSSQ RNRSNYYDAY QAQPLATRSS PRPGQPPKVK
551 SEFNSYSLTG YV

Fusion #D

1 MAAAMADLAE CNIKVMCRFR PLNESEVNRG DKYIAKFQGE DTVVIASKPY
51 AFDRVFQSST SQEQVYNDCA KKIVKDVLEG YNGTIFAYGQ TSSGKTHTME
101 GKLHDPEGMG IIPRIVQDIF NYIYSMDENL EFHIKVSYFE IYLDKIRDLL
151 DVSKTNLSVH EDKNRVPYVK GCTERFVCSP DEVMDTIDEG KSNRHVAVTN
201 MNEHSSRSHS IFLINVKQEN TQTEQKLSGK LYLVDLAGSE KVSKTGAEGA
251 VLDEAKNINK SLSALGNVIS ALAEGSTYVP YRDSKMTRIL QDSLGGNCRT
301 TIVICCSPSS YNESETKSTL LFGQRAKTIK NTVCVNVELT AEQWKKKYEK
351 EKEKNKILRN TIQWLENELN RWRNGETVPI DEQFDKEKAN LEAFTVDKDI
401 TLTNDKPATA IGVIGNFTDA ERRKCEEEIA KLYKQLDDKD EEINQQSQLV
451 EKLKTQMLDQ EELLASTRRD QDNMQAELNR LQAENDASKE EVKEVLQALE
501 ELAVNYDQKS QEVEDKTKEY ELLSDELNQK SATLASIDAE LQKLKEMTNH
551 QKKRAAEMMA SLLKDLAEIG IAVGNNDVKQ PEGTGMIDEE FTVARLYISK
601 MKSEVKTMVK RCKQLESTQT ESNKKMEENE KELAACQLRI SQHEAKIKSL
651 TEYLQNVEQK KRQLEESVDA LSEELVQLRA QEKVHEMEKE HLNKVQTANE
701 VKQAVEQQIQ SHRETHQKQI SSLRDEVEAK AKLITDLQDQ NQKMMLEQER
751 LRVEHEKLKA TDQEKSRKLH ELTVMQDRRE QARQDLKGLE ETVAKELQTL
801 HNLRKLFVQD LATRVKKSAE IDSDDTGGSA AQKQKISFLE NNLEQLTKVH
851 KQLVRDNADL RCELPKLEKR LRATAERVKA LESALKEAKE NASRDRKRYQ
901 QEVDRIKEAG APGSAGSAAG SGMVSKGEED NMASLPATHE LHIFGSINGV
951 DFDMVGQGTG NPNDGYEELN LKSTKGDLQF SPWILVPHIG YGFHQYLPYP
1001 DGMSPFQAAM VDGSGYQVHR TMQFEDGASL TVNYRYTYEG SHIKGEAQVK
1051 GTGFPADGPV MTNSLTAADW CRSKKTYPND KTIISTFKWS YTTGNGKRYR
1101 STARTTYTFA KPMAANYLKN QPMYVFRKTE LKHSKTELNF KEWQKAFTDV
1151 MGMDELYKLE VLFQGPGSSH HHHHSG

Fusion #E

1 MAAAMASVKV AVRVRPMNRR EKDLEAKFII QMEKSKTTIT NLKIPEGGTG
51 DSGRERTKTF TYDFSFYSAD TKSPDYVSQE MVFKTLGTDV VKSAFEGYNA
101 CVFAYGQTGS GKSYTMMGNS GDSGLIPRIC EGLFSRINET TRWDEASFRT
151 EVSYLEIYNE RVRDLLRRKS SKTFNLRVRE HPKEGPYVED LSKHLVQNYG
201 DVEELMDAGN INRTTAATGM NDVSSRSHAI FTIKFTQAKF DSEMPCETVS
251 KIHLVDLAGS ERADATGATG VRLKEGGNIN KSLVTLGNVI SALADLSQDA
301 ANTLAKKKQV FVPYRDSVLT WLLKDSLGGN SKTIMIATIS PADVNYGETL
351 STLRYANRAK NIINKPTINE DANVKLIREL RAEIARLKTL LAQGNQIALL
401 DSPTALSMEE KLQQNEARVQ ELTKEWTNKW NETQNILKEQ TLALRKEGIG
451 VVLDSELPHL IGIDDDLLST GIILYHLKEG QTYVGRDDAS TEQDIVLHGL
501 DLESEHCIFE NIGGTVTLIP LSGSQCSVNG VQIVEATHLN QGAVILLGRT
551 NMFRFNHPKE AAKLREKRKS GLLSSFSLSM TDLSKSRENL SAVMLYNPGL
601 EFERQQREEL EKLESKRKLI EEMEEKQKSD KAELERMQQE VETQRKETEI
651 VQLQIRKQEE SLKRRSFHIE NKLKDLLAEK EKFEEERLRE QQEIELQKKR
701 QEEETFLRVQ EELQRLKELN NNEKAEKFQI FQELDQLQKE KDEQYAKLEL
751 EKKRLEEQEK EQVMLVAHLE EQLREKQEMI QLLRRGEVQW VEEEKRDLEG
801 IRESLLRVKE ARAGGDEDGE ELEKAQLRFF EFKRRQLVKL VNLEKDLVQQ
851 KDILKKEVQE EQEILECLKC EHDKESRLLE KHDESVTDVT EVPQDFEKIK
901 PVEYRLQYKE RQLQYLLQNH LPTLLEEKQR AFEILDRGPL SLDNTLYQVE
951 KEMEEKEEQL AQYQANANQL QKLQATFEFT ANIARQEEKV RKKEKEILES
1001 REKQQREALE RALARLERRH SALQRHSTLG TEIEEQRQKL ASLNSGSREQ
1051 SGLQASLEAE QEALEKDQER LEYEIQQLKQ KIYEVDGVQK DHHGTLEGKV
1101 ASSSLPVSAE KSHLVPLMDA RINAYIEEEV QRRLQDLHRV ISEGCSTSAD
1151 TMKDNEKLHN GTIQRKLKYE RMVSRSLGAN PDDLKDPIKI SIPRYVLCGQ
1201 GKDAHFEFEV KITVLDETWT VFRRYSRFRE MHKTLKLKYA ELAALEFPPK
1251 KLFGNKDERV IAERRSHLEK YLRDFFSVML QSATSPLHIN KVGLTLSKHT
1301 ICEFSPFFKK GVFDYSSHGT GGAPGSAGSA AGSGMVSKGE AVIKEFMRFK
1351 VHMEGSMNGH EFEIEGEGEG RPYEGTQTAK LKVTKGGPLP FSWDILSPQF
1401 MYGSRAFIKH PADIPDYYKQ SFPEGFKWER VMNFEDGGAV TVTQDTSLED
1451 GTLIYKVKLR GTNFPPDGPV MQKKTMGWEA STERLYPEDG VLKGDIKMAL
1501 RLKDGGRYLA DFKTTYKAKK PVQMPGAYNV DRKLDITSHN EDYTVVEQYE
1551 RSEGRHSTGG MDELYKNAPL EVLFQGPGSS MKIEEGKLVI WINGDKGYNG
1601 LAEVGKKFEK DTGIKVTVEH PDKLEEKFPQ VAATGDGPDI IFWAHDRFGG
1651 YAQSGLLAEI TPDKAFQDKL YPFTWDAVRY NGKLIAYPIA VEALSLIYNK
1701 DLLPNPPKTW EEIPALDKEL KAKGKSALMF NLQEPYFTWP LIAADGGYAF
1751 KYENGKYDIK DVGVDNAGAK AGLTFLVDLI KNKHMNADTD YSIAEAAFNK
1801 GETAMTINGP WAWSNIDTSK VNYGVTVLPT FKGQPSKPFV GVLSAGINAA
1851 SPNKELAKEF LENYLLTDEG LEAVNKDKPL GAVALKSYEE ELVKDPRIAA
1901 TMENAQKGEI MPNIPQMSAF WYAVRTAVIN AASGRQTVDE ALKDAQTNSS
1951 SNNNNNNNNN NSG

Fusion #F

1 MAAAAEPRQE FEVMEDHAGT YGLGDRKDQG GYTMHQDQEG DTDAGLKESP
51 LQTPTEDGSE EPGSETSDAK STPTAEDVTA PLVDEGAPGK QAAAQPHTEI
101 PEGTTAEEAG IGDTPSLEDE AAGHVTQARM VSKSKDGTGS DDKKAKGADG
151 KTKIATPRGA APPGQKGQAN ATRIPAKTPP APKTPPSSGE PPKSGDRSGY
201 SSPGSPGTPG SRSRTPSLPT PPTREPKKVA VVRTPPKSPS SAKSRLQTAP
251 VPMPDLKNVK SKIGSTENLK HQPGGGKVQI INKKLDLSNV QSKCGSKDNI
301 KHVPGGGSVQ IVYKPVDLSK VTSKCGSLGN IHHKPGGGQV EVKSEKLDFK
351 DRVQSKIGSL DNITHVPGGG NKKIETHKLT FRENAKAKTD HGAEIVYKSP
401 VVSGDTSPRH LSNVSSTGSI DMVDSPQLAT LADEVSASLA KQGLGAPGSA
451 GSAAGSGMNT PGINLIKEDM RVKVHMEGNV NGHAFVIEGE GKGKPYEGTQ
501 TANLTVKEGA PLPFSYDILT TAVHYGNRVF TKYPEDIPDY FKQSFPEGYS
551 WERTMTFEDK GICTIRSDIS LEGDCFFQNV RFKGTNFPPN GPVMQKKTLK
601 WEPSTEKLHV RDGLLVGNIN MALLLEGGGH YLCDFKTTYK AKKVVQLPDA
651 HFVDHRIEIL GNDSDYNKVK LYEHAVARYS PLPSQVWLEV LFQGPGSSHH
701 HHHHSG

Fusion #G

1 MGHHHHHHSS GRMKIEEGKL VIWINGDKGY NGLAEVGKKF EKDTGIKVTV
51 EHPDKLEEKF PQVAATGDGP DIIFWAHDRF GGYAQSGLLA EITPDKAFQD
101 KLYPFTWDAV RYNGKLIAYP IAVEALSLIY NKDLLPNPPK TWEEIPALDK
151 ELKAKGKSAL MFNLQEPYFT WPLIAADGGY AFKYENGKYD IKDVGVDNAG
201 AKAGLTFLVD LIKNKHMNAD TDYSIAEAAF NKGETAMTIN GPWAWSNIDT
251 SKVNYGVTVL PTFKGQPSKP FVGVLSAGIN AASPNKELAK EFLENYLLTD
301 EGLEAVNKDK PLGAVALKSY EEELVKDPRI AATMENAQKG EIMPNIPQMS
351 AFWYAVRTAV INAASGRQTV DEALKDAQTN SSSNNNNNNN NNNSSGRLEV
401 LFQGPAAAMP VRGDRGFPPR RELSGWLRAP GMEELIWEQY TVTLQKDSKR
451 GFGIAVSGGR DNPHFENGET SIVISDVLPG GPADGLLQEN DRVVMVNGTP
501 MEDVLHSFAV QQLRKSGKVA AIVVKRPRKV QVAALQASPP LDQDDRAFEV
551 MDEFDGRSFR SGYSERSRLN SHGGRSRSWE DSPERGRPHE RARSRERDLS
601 RDRSRGRSLE RGLDQDHART RDRSRGRSLE RGLDHDFGPS RDRDRDRSRG
651 RSIDQDYERA YHRAYDPDYE RAYSPEYRRG ARHDARSRGP RSRSREHPHS
701 RSPSPEPRGR PGPIGVLLMK SRANEEYGLR LGSQIFVKEM TRTGLATKDG
751 NLHEGDIILK INGTVTENMS LTDARKLIEK SRGKLQLVVL RDSQQTLINI
801 PSLNDSDSEI EDISEIESNR SFSPEERRHQ YSDYDYHSSS EKLKERPSSR
851 EDTPSRLSRM GATPTPFKST GDIAGTVVPE TNKEPRYQED PPAPQPKAAP
901 RTFLRPSPED EAIYGPNTKM VRFKKGDSVG LRLAGGNDVG IFVAGIQEGT
951 SAEQEGLQEG DQILKVNTQD FRGLVREDAV LYLLEIPKGE MVTILAQSRA
1001 DVYRDILACG RGDSFFIRSH FECEKETPQS LAFTRGEVFR VVDTLYDGKL
1051 GNWLAVRIGN ELEKGLIPNK SRAEQMASVQ NAQRDNAGDR ADFWRMRGQR
1101 SGVKKNLRKS REDLTAVVSV STKFPAYERV LLREAGFKRP VVLFGPIADI
1151 AMEKLANELP DWFQTAKTEP KDAGSEKSTG VVRLNTVRQI IEQDKHALLD
1201 VTPKAVDLLN YTQWFPIVIF FNPDSRQGVK TMRQRLNPTS NKSSRKLFDQ
1251 ANKLKKTCAH LFTATINLNS ANDSWFGSLK DTIQHQQGEA VWVSEGKMEG
1301 MDDDPEDRMS YLTAMGADYL SCDSRLISDF EDTDGEGGAY TDNELDEPAE
1351 EPLVSSITRS SEPVQHEESI RKPSPEPRAQ MRRAASSDQL RDNSPPPAFK
1401 PEPPKAKTQN KEESYDFSKS YEYKSNPSAV AGNETPGAST KGYPPPVAAK
1451 PTFGRSILKP STPIPPQEGE EVGESSEEQD NAPKSVLGKV KIFEKMDHKA
1501 RLQRMQELQE AQNARIEIAQ KHPDIYAVPI KTHKPDPGTP QHTSSRPPEP
1551 QKAPSRPYQD TRGSYGSDAE EEEYRQQLSE HSKRGYYGQS ARYRDTELGA
1601 PGSSSGRENL YFQGMVSKGE ELFTGVVPIL VELDGDVNGH KFSVSGEGEG
1651 DATYGKLTLK FICTTGKLPV PWPTLVTTLT YGVQCFSRYP DHMKQHDFFK
1701 SAMPEGYVQE RTIFFKDDGN YKTRAEVKFE GDTLVNRIEL KGIDFKEDGN
1751 ILGHKLEYNY NSHNVYIMAD KQKNGIKVNF KIRHNIEDGS VQLADHYQQN
1801 TPIGDGPVLL PDNHYLSTQS KLSKDPNEKR DHMVLLEFVT AAGITLGMDE
1851 LYK

Fusion #H

1 MGSSHHHHHH SSGRMKIEEG KLVIWINGDK GYNGLAEVGK KFEKDTGIKV
51 TVEHPDKLEE KFPQVAATGD GPDIIFWAHD RFGGYAQSGL LAEITPDKAF
101 QDKLYPFTWD AVRYNGKLIA YPIAVEALSL IYNKDLLPNP PKTWEEIPAL
151 DKELKAKGKS ALMFNLQEPY FTWPLIAADG GYAFKYENGK YDIKDVGVDN
201 AGAKAGLTFL VDLIKNKHMN ADTDYSIAEA AFNKGETAMT INGPWAWSNI
251 DTSKVNYGVT VLPTFKGQPS KPFVGVLSAG INAASPNKEL AKEFLENYLL
301 TDEGLEAVNK DKPLGAVALK SYEEELVKDP RIAATMENAQ KGEIMPNIPQ
351 MSAFWYAVRT AVINAASGRQ TVDEALKDAQ TNSSSNNNNN NNNNNSSGRL
401 EVLFQGPMDK DCEMKRTTLD SPLGKLELSG CEQGLHRIIF LGKGTSAADA
451 VEVPAPAAVL GGPEPLMQAT AWLNAYFHQP EAIEEFPVPA LHHPVFQQES
501 FTRQVLWKLL KVVKFGEVIS YSHLAALAGN PAATAAVKTA LSGNPVPILI
551 PCHRVVQGDL DVGGYEGGLA VKEWLLAHEG HRLGKPGLGG SSSGRENLYF
601 QGAAAMPVRG DRGFPPRREL SGWLRAPGME ELIWEQYTVT LQKDSKRGFG
651 IAVSGGRDNP HFENGETSIV ISDVLPGGPA DGLLQENDRV VMVNGTPMED
701 VLHSFAVQQL RKSGKVAAIV VKRPRKVQVA ALQASPPLDQ DDRAFEVMDE
751 FDGRSFRSGY SERSRLNSHG GRSRSWEDSP ERGRPHERAR SRERDLSRDR
801 SRGRSLERGL DQDHARTRDR SRGRSLERGL DHDFGPSRDR DRDRSRGRSI
851 DQDYERAYHR AYDPDYERAY SPEYRRGARH DARSRGPRSR SREHPHSRSP
901 SPEPRGRPGP IGVLLMKSRA NEEYGLRLGS QIFVKEMTRT GLATKDGNLH
951 EGDIILKING TVTENMSLTD ARKLIEKSRG KLQLVVLRDS QQTLINIPSL
1001 NDSDSEIEDI SEIESNRSFS PEERRHQYSD YDYHSSSEKL KERPSSREDT
1051 PSRLSRMGAT PTPFKSTGDI AGTVVPETNK EPRYQEDPPA PQPKAAPRTF
1101 LRPSPEDEAI YGPNTKMVRF KKGDSVGLRL AGGNDVGIFV AGIQEGTSAE
1151 QEGLQEGDQI LKVNTQDFRG LVREDAVLYL LEIPKGEMVT ILAQSRADVY
1201 RDILACGRGD SFFIRSHFEC EKETPQSLAF TRGEVFRVVD TLYDGKLGNW
1251 LAVRIGNELE KGLIPNKSRA EQMASVQNAQ RDNAGDRADF WRMRGQRSGV
1301 KKNLRKSRED LTAVVSVSTK FPAYERVLLR EAGFKRPVVL FGPIADIAME
1351 KLANELPDWF QTAKTEPKDA GSEKSTGVVR LNTVRQIIEQ DKHALLDVTP
1401 KAVDLLNYTQ WFPIVIFFNP DSRQGVKTMR QRLNPTSNKS SRKLFDQANK
1451 LKKTCAHLFT ATINLNSAND SWFGSLKDTI QHQQGEAVWV SEGKMEGMDD
1501 DPEDRMSYLT AMGADYLSCD SRLISDFEDT DGEGGAYTDN ELDEPAEEPL
1551 VSSITRSSEP VQHEESIRKP SPEPRAQMRR AASSDQLRDN SPPPAFKPEP
1601 PKAKTQNKEE SYDFSKSYEY KSNPSAVAGN ETPGASTKGY PPPVAAKPTF
1651 GRSILKPSTP IPPQEGEEVG ESSEEQDNAP KSVLGKVKIF EKMDHKARLQ
1701 RMQELQEAQN ARIEIAQKHP DIYAVPIKTH KPDPGTPQHT SSRPPEPQKA
1751 PSRPYQDTRG SYGSDAEEEE YRQQLSEHSK RGYYGQSARY RDTELGAP

- -

Fusion #I

1 MGSSHHHHHH SSGRMKIEEG KLVIWINGDK GYNGLAEVGK KFEKDTGIKV
51 TVEHPDKLEE KFPQVAATGD GPDIIFWAHD RFGGYAQSGL LAEITPDKAF
101 QDKLYPFTWD AVRYNGKLIA YPIAVEALSL IYNKDLLPNP PKTWEEIPAL
151 DKELKAKGKS ALMFNLQEPY FTWPLIAADG GYAFKYENGK YDIKDVGVDN
201 AGAKAGLTFL VDLIKNKHMN ADTDYSIAEA AFNKGETAMT INGPWAWSNI
251 DTSKVNYGVT VLPTFKGQPS KPFVGVLSAG INAASPNKEL AKEFLENYLL
301 TDEGLEAVNK DKPLGAVALK SYEEELVKDP RIAATMENAQ KGEIMPNIPQ
351 MSAFWYAVRT AVINAASGRQ TVDEALKDAQ TNSSSNNNNN NNNNNSSGRL
401 EVLFQGPMVS KGEEDNMAII KEFMRFKVHM EGSVNGHEFE IEGEGEGRPY
451 EGTQTAKLKV TKGGPLPFAW DILSPQFMYG SKAYVKHPAD IPDYLKLSFP
501 EGFKWERVMN FEDGGVVTVT QDSSLQDGEF IYKVKLRGTN FPSDGPVMQK
551 KTMGWEASSE RMYPEDGALK GEIKQRLKLK DGGHYDAEVK TTYKAKKPVQ
601 LPGAYNVNIK LDITSHNEDY TIVEQYERAE GRHSTGGMDE LYKGSAGSAA
651 GSGAAAMPVR GDRGFPPRRE LSGWLRAPGM EELIWEQYTV TLQKDSKRGF
701 GIAVSGGRDN PHFENGETSI VISDVLPGGP ADGLLQENDR VVMVNGTPME
751 DVLHSFAVQQ LRKSGKVAAI VVKRPRKVQV AALQASPPLD QDDRAFEVMD
801 EFDGRSFRSG YSERSRLNSH GGRSRSWEDS PERGRPHERA RSRERDLSRD
851 RSRGRSLERG LDQDHARTRD RSRGRSLERG LDHDFGPSRD RDRDRSRGRS
901 IDQDYERAYH RAYDPDYERA YSPEYRRGAR HDARSRGPRS RSREHPHSRS
951 PSPEPRGRPG PIGVLLMKSR ANEEYGLRLG SQIFVKEMTR TGLATKDGNL
1001 HEGDIILKIN GTVTENMSLT DARKLIEKSR GKLQLVVLRD SQQTLINIPS
1051 LNDSDSEIED ISEIESNRSF SPEERRHQYS DYDYHSSSEK LKERPSSRED
1101 TPSRLSRMGA TPTPFKSTGD IAGTVVPETN KEPRYQEDPP APQPKAAPRT
1151 FLRPSPEDEA IYGPNTKMVR FKKGDSVGLR LAGGNDVGIF VAGIQEGTSA
1201 EQEGLQEGDQ ILKVNTQDFR GLVREDAVLY LLEIPKGEMV TILAQSRADV
1251 YRDILACGRG DSFFIRSHFE CEKETPQSLA FTRGEVFRVV DTLYDGKLGN
1301 WLAVRIGNEL EKGLIPNKSR AEQMASVQNA QRDNAGDRAD FWRMRGQRSG
1351 VKKNLRKSRE DLTAVVSVST KFPAYERVLL REAGFKRPVV LFGPIADIAM
1401 EKLANELPDW FQTAKTEPKD AGSEKSTGVV RLNTVRQIIE QDKHALLDVT
1451 PKAVDLLNYT QWFPIVIFFN PDSRQGVKTM RQRLNPTSNK SSRKLFDQAN
1501 KLKKTCAHLF TATINLNSAN DSWFGSLKDT IQHQQGEAVW VSEGKMEGMD
1551 DDPEDRMSYL TAMGADYLSC DSRLISDFED TDGEGGAYTD NELDEPAEEP
1601 LVSSITRSSE PVQHEESIRK PSPEPRAQMR RAASSDQLRD NSPPPAFKPE
1651 PPKAKTQNKE ESYDFSKSYE YKSNPSAVAG NETPGASTKG YPPPVAAKPT
1701 FGRSILKPST PIPPQEGEEV GESSEEQDNA PKSVLGKVKI FEKMDHKARL
1751 QRMQELQEAQ NARIEIAQKH PDIYAVPIKT HKPDPGTPQH TSSRPPEPQK
1801 APSRPYQDTR GSYGSDAEEE EYRQQLSEHS KRGYYGQSAR YRDTELGAP

Supplementary Figure S4. Peptide mapping of FP-fusions by mass spectrometry. Peptides detected by ms-proteomics analysis on the tryptic digests of each FP-fusion are shown in red.

.

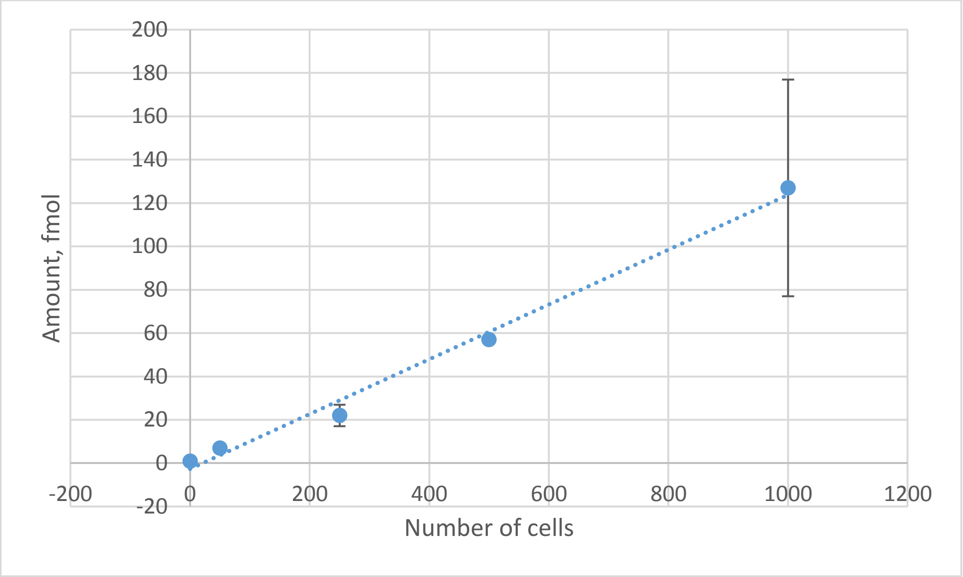

Supplementary Figure S5. Absolute quantification of EGFP in stably transfected HeLa cells with qFP-8 standard. Cell lysate of HeLa stably expressing mEGFP (Suppl. Table S4, #2) was separated by SDS PAGE, FP band digested with trypsin and equivalent of 1000, 500, 250 and 50 cells analyzed by mass spectrometry using qFP-8 peptide proxies.

A

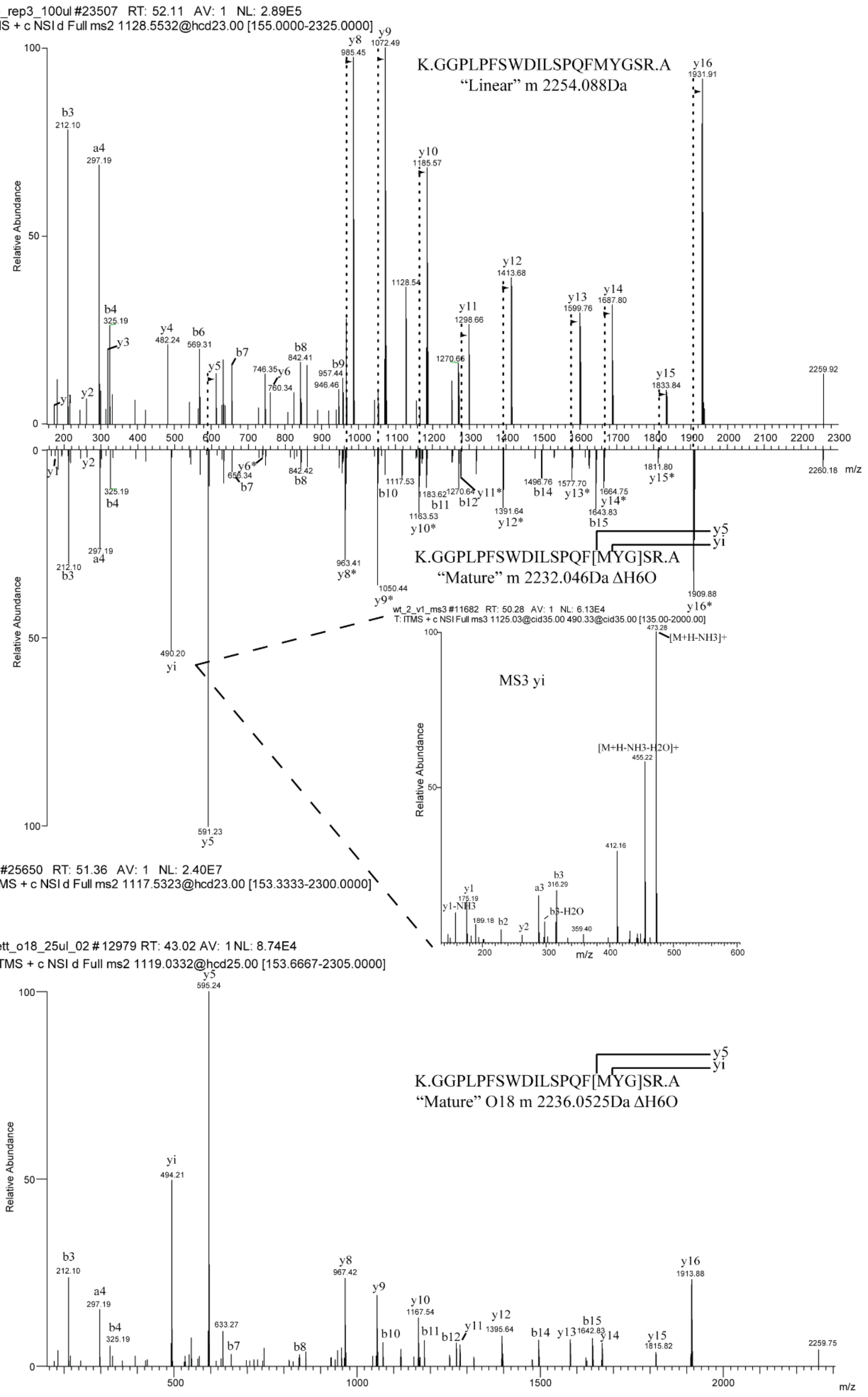
sissi_6_rep3_100ul #23507 RT: 52.11 AV: 1 NL: 2.89E5
T: FTMS + c NSI d Full ms2 1128.5532@hcd23.00 [155.0000-2325.0000]
K.GGPLPFSWDILSPQFMYGSR.A
"Linear" m 2254.088Da
K.GGPLPFSWDILSPQF[MYG]SR.A
"Mature" m 2232.046Da ΔH6O
wt_2_v1_ms3 #11682 RT: 50.28 AV: 1 NL: 6.13E4
T: ITMS + c NSI Full ms3 1125.03@cid35.00 490.33@cid35.00 [135.00-2000.00]
MS3 yi
[M+H-NH3]+
[M+H-NH3-H2O]+
s_w2 #25650 RT: 51.36 AV: 1 NL: 2.40E7
T: FTMS + c NSI d Full ms2 1117.5323@hcd23.00 [153.3333-2300.0000]
scarlett_o18_25ul_02 # 12979 RT: 43.02 AV: 1 NL: 8.74E4
T: FTMS + c NSI d Full ms2 1119.0332@hcd25.00 [153.6667-2305.0000]
K.GGPLPFSWDILSPQF[MYG]SR.A
"Mature" O18 m 2236.0525Da ΔH6O
Relative Abundance
m/z

B

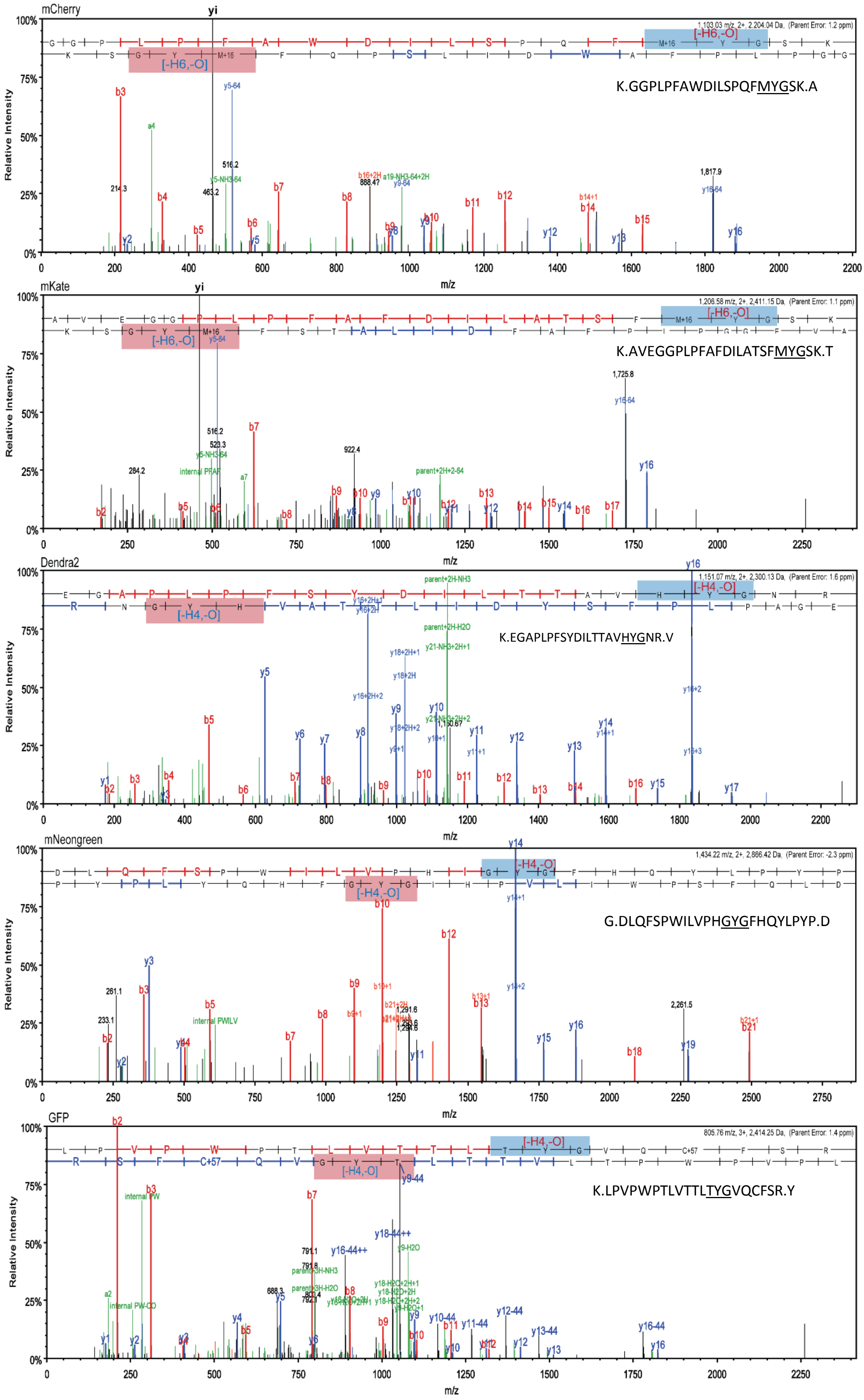
mCherry
K.GGPLPFAWDILSPQFMYGSK.A
[-H6,-O]
1,103.03 m/z, 2+, 2,204.04 Da, (Parent Error: 1.2 ppm)
mKate
K.AVEGGPLPFAFDILATSFMYGSK.T
[-H6,-O]
1,206.58 m/z, 2+, 2,411.15 Da, (Parent Error: 1.1 ppm)
Dendra2
K.EGAPLPFSYDILTTAVHYGNR.V
[-H4,-O]
1,151.07 m/z, 2+, 2,300.13 Da, (Parent Error: 1.6 ppm)
mNeongreen
G.DLQFSPWILVPHGYGFHQYLPYP.D
[-H4,-O]
1,434.22 m/z, 2+, 2,866.42 Da, (Parent Error: -2.3 ppm)
GFP
K.LPVPWPTLVTTLTYGVQCFSR.Y
[-H4,-O]
805.76 m/z, 3+, 2,414.25 Da, (Parent Error: 1.4 ppm)
Relative Intensity
m/z

Supplementary Figure S6. Fragmentation spectra of chromophore-containing peptides from red- and green-type FPs

A. Fragmentation spectra of mScarlet-I peptide with linear and mature (cyclic) chromophore tripeptide …MYG… Abundant fragment y_i is formed by cleavage of internal chemical bond within the chromophore cycle. The identity of yi was confirmed by MS3 fragmentation (insert) and meatabolic labeling of C-terminal arginine residue with $H_2^{18}O$ (lower panel, all y-ions show characteristic 4Da mass shift). Note that, upon digestion with trypsin in $H_2^{18}O$ water arginine residues exchange both oxygene atoms within their C-terminal carboxyl group.
B. Fragmentation spectra of peptides comprising mature (cyclic) chromophore detected in red-type (mKate2, mCherry) and green-type FPs (Dendra2, mNeonGreen, EGFP). Signature of two abundant fragments – yi (resulted from cleavage of internal bound withing cyclic chromophore) and y5 - is characteristic for red type of mature chromophore. GFP peptide with cyclic chromophore …TYG… shows characteristic series of chromophore-containing y-ions with the $\Delta m=44.026$ Da. Chromophore sequence is highlighted in blue and red for y- and b-ions, respectively.

A

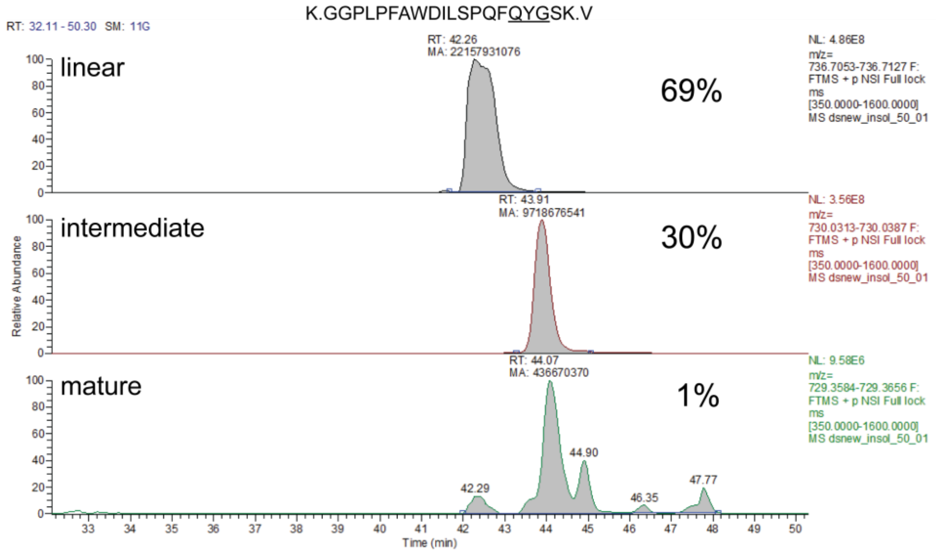

B

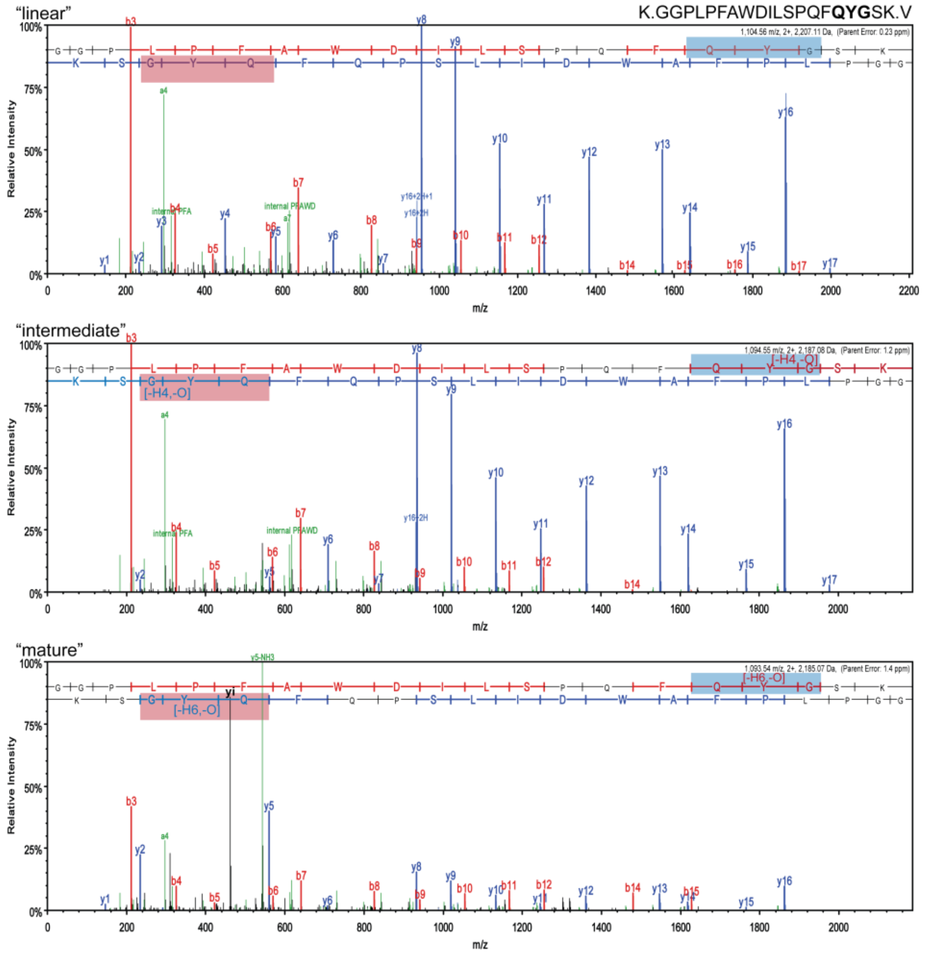

Supplementary Figure S7. Chromophore-containing peptides detected in red-type FP dsRed-express by mass spectrometry. Extracted Ion Chromatograms (XICs) (**Panel A**) and fragmentation spectra (**Panel B**) of dsRed-express peptide GGPLPFAWDILSPQFQYGSK comprising linear, intermediate and mature cyclic forms of …QYG… chromophore tripeptide.

A

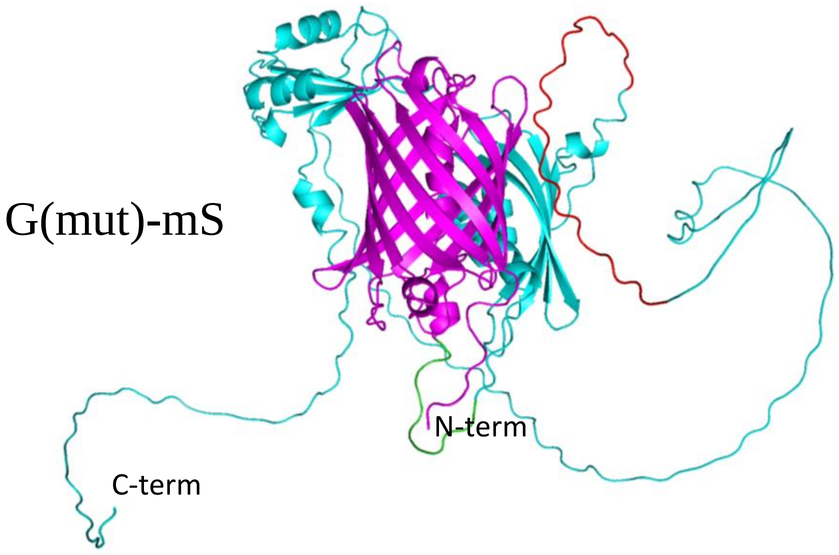

B

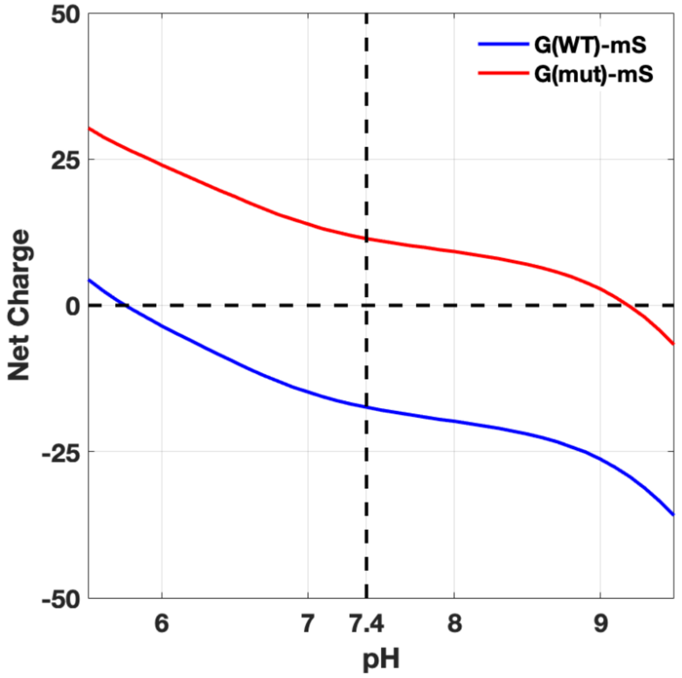

Supplementary Figure S8. Removal of glutamic acid residues from intrinsically disordered region changed formal net charge of the mScarlet-tagged protein G3BP1. **A**: 3D structure of the G(mut)-mS where glutamic acid residues are removed from the intrinsically disordered region (IDR). G3BP1 is shown in magenta, with the IDR (red) after removal of glutamic acid residues and short spacer sequence (green); fused red FP mScarlet-I is shown in pink. **B**: Net formal charge to pH curve for G(WT)_mS and G(mut)-mS.

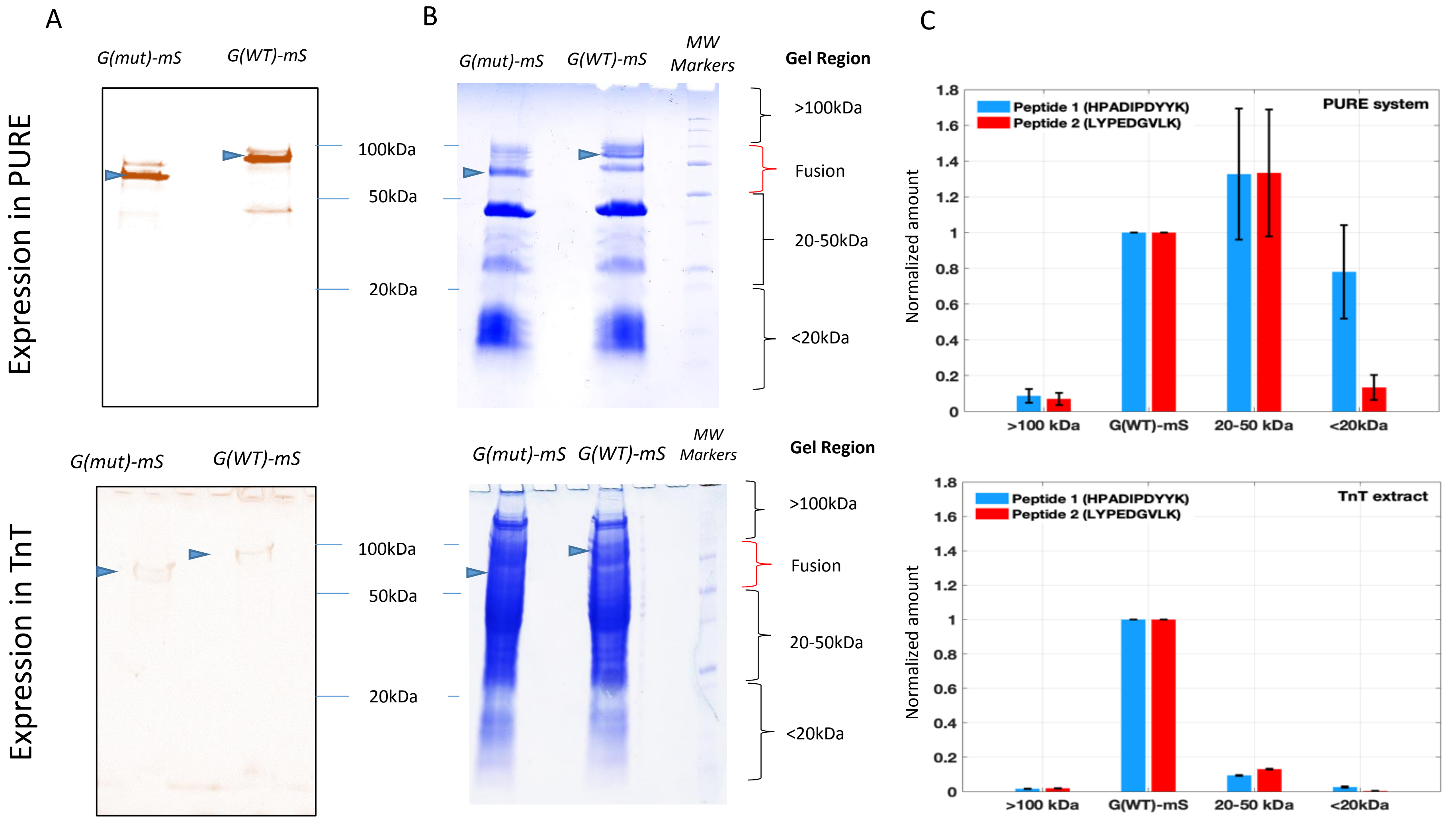

Supplementary Figure S9. Analysis of short products of expression. G(WT)-mS and G(mut)-mS were expressed for 4h in PURE and TnT cell free systems and aliquots of extracts separated on SDS PAGE under non-reducing conditions with no heating. **A:** fluorescence imaging of gel-separated extracts at Cy3 channel relevant to the mScarlet-I fluorescence; **B:** Corresponding SDS gels visualized by Coomassie. The position of full-length product is designated with arrow. Regions excised from the G(WT)-mS gel lane for quantification of FP peptides are indicated on the right side. **C:** Amount of mScarlet peptides 76-85 HPADIPDYYK and 151-159 LYPEDGVLK quantified using qFP-8 in G(WT)-mS at four gel regions of the G(WT)-mS gel lane marked on panel B.

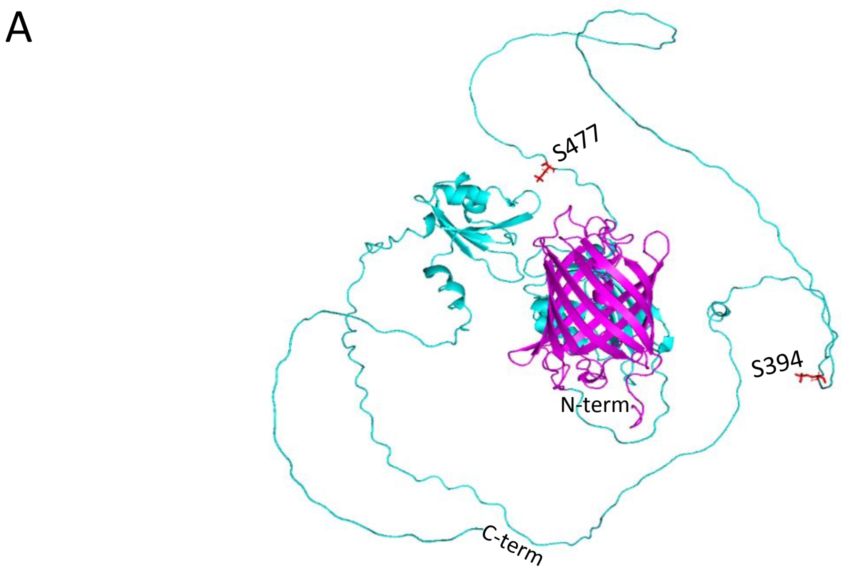

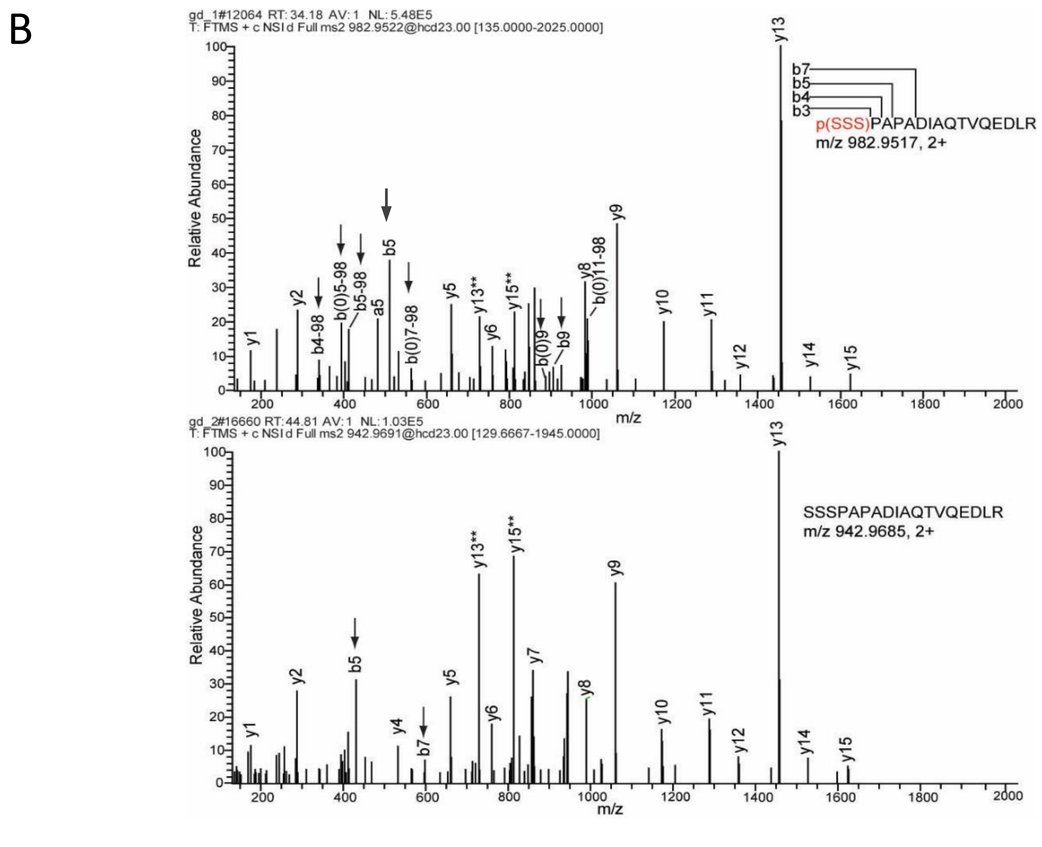

Supplementary Figure S10. Detection of serine phosphorylation in G(WT)-mS expressed in TnT cell-free system by mass spectrometry. **A:** 3D structure of G(WT)-mS. Position of phosphorylated serine residues S149 and S232 (393 and 477 in G(WT)-mS sequence, respectively) are shown in red. **B:** Fragmentation spectra of the mono-phosphorylated peptide (230)SSSPAPADIAQTVQEDLR(248) (upper panel) and unmodified (lower panel). Mono-phosphorylated serine cluster 230-233 is marked in red; the corresponding b-ions are designated with arrows on the spectrum.

A

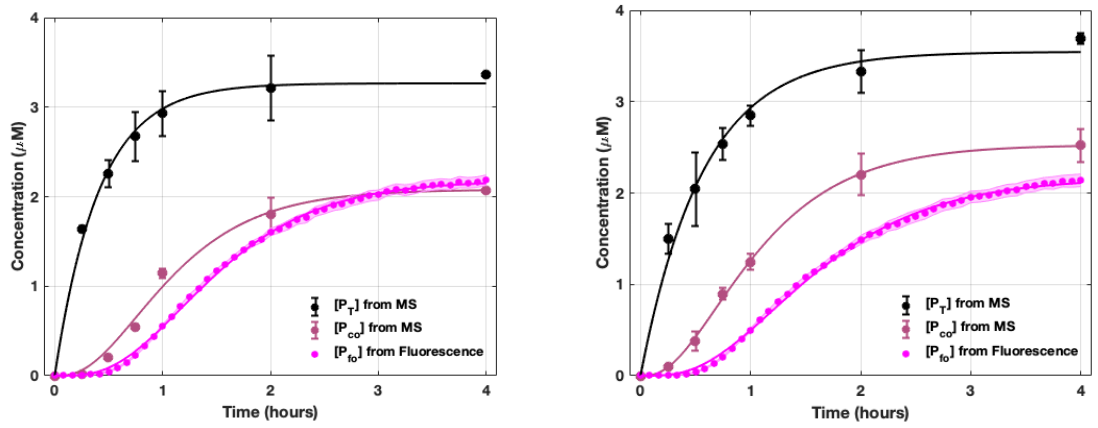

B

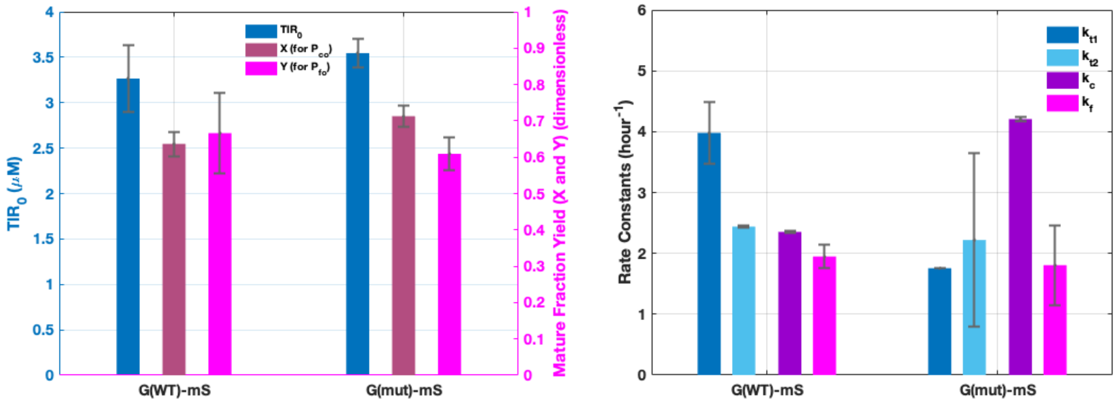

C

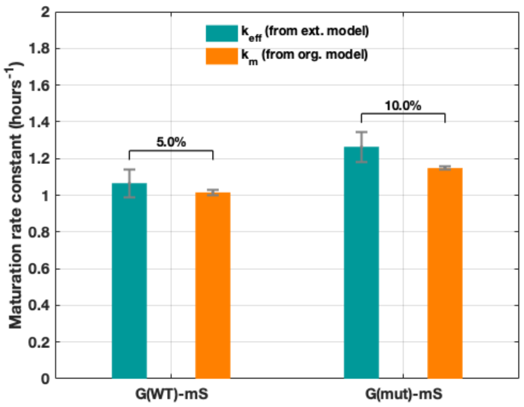

Supplementary Figure S11. Extended model. **A**: Fits to the extended model for G(WT)-mS (left) and G(mut)-mS (right); **B**: Fitting parameters from the extended model; **C**: Comparison between $k_{eff} = (k_c^{-1} + k_f^{-1})^{-1}$ from the extended model and k_m from the original model highlighting minimal percentage differences.

A

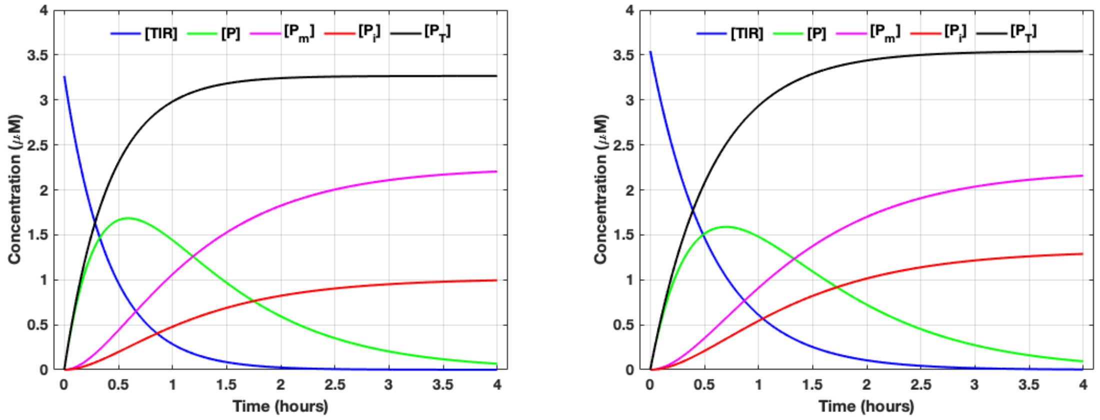

B

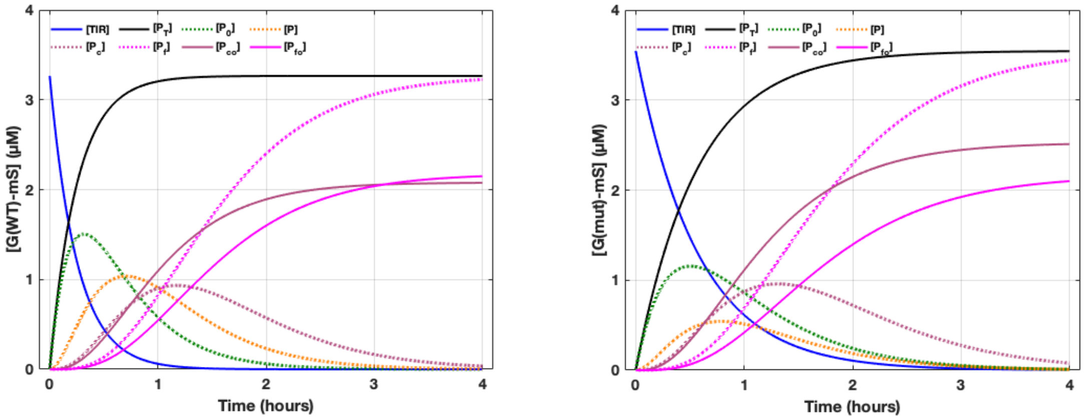

Supplementary Figure S12. Simulations of hidden variables using the original and extended models.

A. Simulations using the original model for G(WT)-mS (left) and G(mut)-mS (right) showing the kinetic traces for translation nutrient TlR, immature intermediate P, mature protein P_m, dark fraction that never matured, P_i and the total protein P_T.

B. Simulations using the extended model for G(WT)-mS (left) and G(mut)-mS (right) showing the kinetic traces for translation nutrient TlR, precursor P_0, immature intermediate P, total chromophore mature fraction P_c, fraction of the chromophore mature fraction actually observed P_{co} by mass spectrometry, total fully mature folded fraction P_f, fraction of the fully mature folded fraction actually observed P_{fo} by fluorescence spectroscopy, and the total protein P_T as observed by mass spectrometry.

Supplementary Table S1

List of Fluorescent Proteins, Self-labelling Tags and their Fusions

N	Name of FP or self-labelling tag	AccN[1]	FP type/ Chromophore tripeptide	MW, kDa	N of quantotypic peptides[2]	FP-fusion		
						code	Fused protein gene name	MW, kDa
Fluorescent Proteins								
1	mKate2	DBB08	Basic far-red FP MYG	26	3	#A	Spd2	158
2	mScarlet-I	6VVTK[3]	Basic RFP MYG	26	4	#E	Kif16B, MBP	230
3	mCherry	ZERB6	Basic RFP MYG	26	3	#I	TJP2, MBP	207
4	mNeonGeen	ZRKRV	Basic yellow-green FP GYG	26	3(1)	#D	KIF5B	134
5	mEGFP	QKFJN	Basic green FP TYG	26	2(1)	#G	TJP2, MBP	207
						#J[6]	NMY2	280
6	Dendra2	GE6KO	Photoconvertible red-to-green FP HYG	26	5	#F	MAPT 471-756 fragment	75
7	Venus	YUJWJ	Basic YFP GYG	26	2(1)[4]	-	-	-
8	TagRFP	S4HC8	Basic orange-red FP MYG	26	2[4]	-	-	-
9	dsRed-Express	DCYCK	Basic FRP QYG	26	2[4]	-	-	-
Non-fluorescent self-labelling proteins								
10	SNAP-Tag	3kzy	-	19	3	#H	TJP2, MBP	200
						#B	FUS, MBP	119
						#J[5]	NMY2	280
11	HaloTag	6U32_A	-	34	3	#C	CLDN2	62

1- From www.fpbase.org or PDB
2- Number of Met-containing peptides is in brackets
3- In-house version of the sequence is missing Val at the position 2
4- Venus, TagRFP and dsRed-Exress sequences share peptides identical with mEGFP, mKate2 and mCherry proxies respectively
5- #J is a double-tagged FP-Fusion, with SNAP-Tag on N-terminus and EGFP on C-terminus

Supplementary Table S2

Peptide proxies included in qFP-8 chimeric standard protein

N	Origin	Peptide Sequence	m/z light form		m/z heavy form[1]	
			+2	+3	+2	+3
Fluorescent proteins and self-labelling protein tags						
1	NeonGreen	YTYEGSHIK	549.266	366.514	552.278	368.521
2		TIISTFK	405.242		408.253	
3		WSYTTGNGK	507.238		510.249	
4		TMQFEDGASLTVNYR[2]	866.404	583.270	871.969	586.607
5	eGFP	FSVSGEGEGDATYGK	752.334		755.344	
6		FEGDTLVNR	525.764		530.769	
7		SAMPEGYVQER[2]	633.793		638.798	
8	Dendra2	YPEDIPDYFK	643.801		646.811	
9		QSFPEGYSWER	693.310		698.315	
10		VVQLPDAHFVDHR		511.604		514.941
11		IEILGNDSDYNK	690.836		693.847	
12		LYEHAVAR	479.759		484.764	
13	mScarlet-I	HPADIPDYYK	609.793	406.865	612.804	408.872
14		QSFPEGFK	470.232		473.243	
		QSFPEGFKWER		470.898		476.241
15		LYPEDGVLK	517.282		520.293	
16	mCherry	HPADIPDYLK	584.804	390.205	587.814	392.212
17		LSFPEGFK	462.745		465.756	
18		LDITSHNEDYTIVEQYER		742.350		745.687
19	mKate	TFINHTQGIPDFFK	555.619	832.925	557.626	834.932
20		QSFPEGFTWER	692.320		697.326	
21		TLGWEASTETLYPADGGLEGR	1112.032	741.690	1117.037	745.027
		AVEGGPLPFAFDILATSFMYGSK[3]				
22	SNAP-Tag	TTLDSPLGK	466.258		469.268	
23		FGEVISYSHLAALAGNPAATAAVK		786.753		788.760
24		VVQGDLDVGGYEGGLAVK	888.462	592.644	891.472	594.651
25	HaloTag	NIIPHVAPTHR	418.907		422.243	
26		LLFWGTPGVLIPPAEAAR	954.541	636.696	959.545	640.033
27		AVDIGPGLNLLQEDNPDLIGSEIAR	1310.185	873.792		877.129
BSA Reference peptides						
1	BSA	DAFLGSFLYEYSR	784.375		789.380	
2		HLVDEPQNLIK	653.362		656.373	
3		LGEYGFQNALIVR	740.401		745.406	
4		LVNELTEFAK	582.319		585.330	
5		YLYEIAR	464.250		469.255	

[1] - metabolic labelled with $^{13}C_6{}^{15}N_4$-Arg and $^{13}C_6$-Lys

[2] – peptides comprising Methionine are not suitable for quantification if chimeric standard is digested separately and spiked into analyte prior ms-analysis

[3] – chromophore-containing peptide is observed in multiple forms depending on maturation state of the chromophore-forming triad (underlined)

Supplementary Table S3

MS-based approaches for absolute quantification of proteins using peptide references and spiked protein standards

	Approach	Reference peptides		Spiked protein standard	Application	Peptides compared during quantification	Ref
		Required features	Selected from				
A	Targeted quantification	quantotypic peptides	FP-tag of the FP-fusion	qFP-8	Targeted	Related peptides from FP (native) and qFP-8 (labelled)	*(34)**
B	MBAQ	“best 3”: 3 peptides with most concordant abundance	Full length sequence	FUGIS	Untargeted	Median abundance of best 3 peptides in fusion vs all FUGIS peptides	*(40)*
C	Top3-Hi	“top 3”: 3 most abundant peptides	Full length sequence	any protein with known amount (e.g.BSA)	Untargeted	Average abundance of top 3 peptides in fusion vs reference protein	*(41)*

*The workflow for this work was adapted from *(34)*

Supplementary Table S4

Amount of FPs-fusions ##A-J quantified using peptide proxies of the qFP-8 chimeric standard

FP-Fusion code[1]	#A	#B	#C	#D	#E	#F	#G	#H	#I	#J[2]	
FP/tag	mKate2	SNAP-tag	HaloTag	mNeonGreen	mScarlet-I	Dendra2	mEGFP	Snap-tag	mCherry	Snap-tag	mEGFP
Calculated amount, pmol[3]	0.620	0.328	1.179	0.293	0.104	0.019	1.526	1.598	2.086	0.170	0.198
CV, %	14	15	5	3	18	3	5	5	5	16	23
CV for individual peptide proxies, %	15	11	18	10	11	23	19	7	15	15	24

1 - see Supplementary Dataset S1 for details

2 – #J is a double-tagged fusion carrying EGFP and Snap-Tag on its N- and C-termini respectively.

3 – in the gel bands corresponding to the full length of the FP-fusion. Equal volume aliquots of lysate of cells expressing an FP-fusion were separated by 1D SDS PAGE prior analysis.

Supplementary Table S5

Examples of FP amounts in stably transfected cells quantified using qFP-8 standard

N	FP	ID (FPbase)	Expressed in (cell type)	FP concentration quantified using qFP-8	FP amount per cell[2]	Cell line proprietor[3]
1	mEGFP	QKFJN	HeLa[1]	81.7 µM	147 amol	*A*
2	mEGFP	QKFJN	HeLa[1]	212.1µM	382 amol	*A*
3	Venus	YUJWJ	HCT116	3.9µM	2 amol	*B*
4	TagRFP	S4HC8	HCT116	17.7µM	9 amol	*B*
5	mScarlet-I	6VVTK	E.coli	5.2mM	5.7 amol	*C*
6	mKate2	DBB08	E.coli	0.6mM	0.6 amol	*C*
7	mCherry	ZERB6	E.coli	0.8mM	0.9 amol	*C*

[1] - two independently prepared cell lanes
[2] - recalculated from FP concentration
[3] – A: Technology Development Studio; B: Dr. M.Sarov (Genome Engineering Facility); C: Protein Biochemistry Facility (all at MPI CBG, Dresden)

Supplementary Table S6

Abundance of chromophore-containing peptides in red FP mScarlet and dsRed-express

**FP**	**Chromophore containing peptide[3]**	**Chromophore maturation form[4]**	**m/z**	**Peptide abundance (XIC)**
mScarlet-I[1]	GGPLPFSWDILSPQ*MYG*SR	linear	757.7017	4.99e8
		intermediate	751.0268	8.21e9
		mature	750.3548	2.23e10
mScarlet mutant M190[2]	GGPLPFSWDILSPQ*MYG*SR	linear	757.7017	2.05e10
		intermediate	751.0268	1.48e8
		mature	750.3548	5.38e8
dsRED-express[1]	GGPLPFAWDILSPQF*QYG*SK	linear	736.7090	2.27e10
		intermediate	730.0350	9.73e9
		mature	729.3620	1.58e7

[1] - as in Supplementary Table S1
[2] – non-fluorescent mutant described in *(49)*
[3] – chromophore tripeptide is shown in Italic, cyclic forms are underlined
[4] –as described in *(50)*

Supplementary table S7

Phosphorylation status of the G3BP1 peptide SSSPAPADIAQTVQEDLR detected by mass spectrometry in G(WT)-mS and G(mut)-mS expressed in TnT and PURE cell free expression systems

FP-fusion	Peptide SSSPAPADIAQTVQEDLR, as detected by mass spectrometry[1]	
	With phosphorylated serine[2]	Unmodified
**Expressed in insect cell line SF9 (control)**		
**G(mut)-mS**	gd_1.12064.12064.2.dta	gd_1.17063.17063.2.dta
**Expressed in PURExpress cell-free system**		
**G(WT)-mS**	n/d	a_1pmolbsa_0_1fp21_pw_inf_1_dda_50ul_1_.25193.25193.2.dta
**G(mut)-mS**	n/d	e_inf_50ul_0_1_fp21_1pmolbsa_dda.16579.16579.2.dta
**Expressed in TnT®T7 cell-free system**		
**G(WT)-mS**	a_1pmolbsa_0_1fp21_i_inf_1_dda_50ul_1.17350.17350.2.dta	a_1pmolbsa_0_1fp21_i_inf_1_dda_50ul_1.16627.16627.2.dta
**G(mut)-mS**	a_inf_50ul_02_20220418083554.23082.23082.2.dta	a_inf_50ul_02_20220418083554.22049.22049.2.dta

[1] ID of the best MS2 spectra; n/d – not detected
[2] the peptide was detected only monophosphorylated by one of Serines
